# Degradation of Antiviral Drug Favipiravir Using UV, UV/H_2_O_2_, and Photocatalysis with Co-Doped ZnS Quantum Dots: Operational Parameters, Kinetic Studies, and Toxicity Assessment

**DOI:** 10.1021/acs.langmuir.4c03639

**Published:** 2025-03-05

**Authors:** Bahriye Eryildiz-Yesir, Hale Ozgun, Mustafa Evren Ersahin, Hamid Reza Rajabi, Vahid Vatanpour, Ismail Koyuncu

**Affiliations:** a Environmental Engineering Department, Istanbul Technical University, Maslak, Istanbul 34469, Turkey; b National Research Center on Membrane Technologies, Istanbul Technical University, Maslak, Istanbul 34469, Turkey; c Chemistry Department, Yasouj University, Yasouj 75918-74831, Iran; d Department of Applied Chemistry, Faculty of Chemistry, Kharazmi University, Tehran 15719-14911, Iran

## Abstract

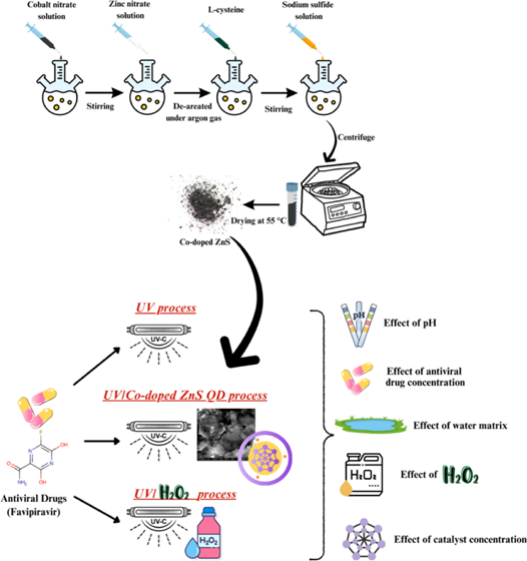

Antiviral drugs, in particular those used to treat COVID-19, have been categorized as emerging pollutants in recent years due to their persistent presence in water/wastewater. They have been identified in environmental matrices all over the world, proving that current treatment methods cannot eliminate them from water/wastewater. In this study, the degradation of favipiravir (FAV) and ecotoxicity changes in different water matrices were investigated using the UV, UV/H_2_O_2_, and UV/Co-doped ZnS quantum dots (QDs) photocatalytic processes. The effects of initial FAV concentration, water matrices, pH, H_2_O_2_ concentration, and catalyst amount on the FAV degradation rate were evaluated. Initial FAV concentrations (50, 100, and 150 μg/L) have a slight effect on the FAV degradation rate. The matrix composition significantly reduced the degradation rates and efficiencies for the UV, UV/H_2_O_2_, and UV/Co-doped ZnS QDs processes in the following order: wastewater treatment plant (WWTP) effluent > tap water (TW) > distilled water (DW). The pH (4.0, 5.0, 7.0, and 9.0) had a remarkable effect on the degradation rate in all processes. The degradation rate enhanced from 86.3 to 98.1% with increasing pH from 4.0 to 9.0 due to increasing the ionization degree of FAV in the UV process. The maximum FAV degradation rate was obtained at pH 7.0 with removal efficiencies of 93.9 and 100% in UV/H_2_O_2_ and UV/Co-doped ZnS QDs processes, respectively. Transformation products of FAV were determined in UV and UV/Co-doped ZnS QDs processes. The toxicity to algae increased with increasing FAV concentrations from 50 to 150 μg/L in distilled water. Growth inhibition rates for 50, 100, and 150 μg/L FAV concentrations were 15.1, 33.3, and 36.3% at 96 h, respectively, without any treatment. After 60 min of the UV process, growth inhibition decreased below 0.5% regardless of concentration. Overall, the UV/Co-doped ZnS QDs process is effective in degrading FAV in all aqueous matrices; however, an initial treatment step is required to remove natural organic matter from actual matrices.

## 1 Introduction

Millions of people (>750 million confirmed cases and ∼7 million deaths) have been affected thus far by the global pandemic known as the coronavirus (COVID-19) by severe acute respiratory syndrome-related coronavirus (SARS-CoV-2), which has spread around the world.^1,2^ Since there are no recognized therapeutic drugs that specifically target the treatment of SARS-CoV-2, lopinavir, favipiravir, remdesivir, azithromycin, hydroxychloroquine, chloroquine, papaverine, nitazoxanide, camostat, nafamostat, ivermectin, mefloquine, darunavir, and ritonavir have been investigated as therapeutic agents to treat COVID-19 patients.^3,4^ Especially, favipiravir was used for the treatment of COVID-19 in several countries such as Russia, Japan, Ukraine, Italy, Uzbekistan, and Turkey.^5^

Favipiravir (FAV) is a pyrazine carboxamide derivative first investigated for the treatment of influenza virus.^6^ Favipiravir has a significant impact on the treatment of COVID-19 and has few negative effects on human health. Favipiravir usage has considerably increased in response to the ongoing COVID-19 pandemic.^7^ Its half-life in the human body is only 2.5–5 h, and the kidneys quickly eliminate it in the hydroxylated form, which is subsequently disposed of in domestic or hospital wastewater.^8^ Favipiravir has a very low degradation rate in the activated sludge process due to its resistance to biodegradation. Conventional wastewater treatment plants have been shown to have a low removal efficiency (less than 20%) in eliminating FAV and its metabolites. Due to these characteristics, FAV was released into the environment. As a result, a substantial concentration (hundreds–thousands ng/L) of FAV and its metabolites has been detected in water as a new emergent concern pollutant, posing a significant ecotoxicological risk.^4,9^ However, there are few studies about the degradation and ecotoxicity of FAV from water/wastewater.^7,8,10^ Therefore, it is imperative to develop efficient and advanced treatment technologies to prevent the health and environmental risks linked to increasing usage of antiviral drugs.

Advanced oxidation processes (AOPs) including photolysis,^11^ ozonation,^12^ photocatalysis,^13^ electrochemical advanced oxidation,^10^ and adsorption^14^ have been widely used for removal of pharmaceutical active compounds (PhACs) including antiviral drugs. Photolysis, including direct, indirect, and self-sensitized photolysis, is an effective method for antiviral drugs to degrade in aquatic environments.^11^ Especially, UV-based AOPs offer various advantages including no transfer of pollutants to another phase as in chemical and biological processes, considerably effective disinfection of pathogens in water and fast reaction rates that are increasing their popularity.^15^ The photolysis efficiency for the antiviral drug removal can be affected by pH, the initial concentration of the antiviral drug, light source, and chemical characteristics of the water/wastewater.^11,16,17^ Also, photocatalysis has attracted considerable attention because of its outstanding catalytic efficiency, capacity to operate under ambient conditions, environmentally friendly characteristics, and nonerosive properties.^18−21^

There are several photocatalyts such as ZnO, Fe_2_O_3_, SnO_2_, ZnS, WO_3_, CeO_2_, CdS, TiO_2_, and quantum dots (QDs) in the literature.^22^ However, quantum dots (QDs) have gained significant attention as efficient and innovative nanophotocatalysts for pollutant removal. Their significance arises from attributes such as elevated quantum yields, high extinction coefficients, reduced photobleaching, narrow and size-tunable emissions, broad absorption spectra, and unique optical and electronic properties distinct from bulk semiconductors.^23^ Among semiconductor QDs, zinc sulfide (ZnS) QDs have attracted attention due to their inherent properties, including high electronic mobility, thermal stability, water insolubility, nontoxic nature, and low cost.^24^ Also, ZnS nanophotocatalysts possess outstanding catalytic characteristics, attributed to the generation of electron–hole pairs and the strongly negative reduction potential of the excited electrons.^25^ The inclusion of transition metal ions such as Fe, Co, and Ni, which display magnetic behavior at room temperature, can introduce magnetic properties to the ZnS QDs, thereby enhancing the host-diluted magnetic semiconductor (DMS) system. The Co ion stands out, particularly due to its high Curie temperature (1388 K) and its ability to exhibit ferromagnetism at room temperature.^26^

The objectives of the present study are (1) to examine the degradation kinetics of FAV during UV, UV/H_2_O_2_, and UV/Co-doped ZnS QDs processes; (2) to investigate the effects of initial FAV concentration in UV photolysis, pH, and water matrices (distilled water, tap water, and wastewater treatment plant effluent) on the degradation kinetics of FAV in the UV, UV/H_2_O_2_, and UV/Co-doped ZnS QDs processes; (3) to determine transformation products of FAV in the UV and UV/Co-doped ZnS QDs processes; (4) to specify energy consumption as a function COD and TOC removal; (5) to evaluate the ecotoxicity risks of FAV using algae with treated solutions that included different initial FAV concentrations. To our knowledge, this is the first study to investigate FAV degradation in the UV, UV/H_2_O_2_, and UV/Co-doped ZnS QDs processes and the ecotoxicological impact of FAV.

## 2 Materials and Methods

### 2.1 Chemicals and Reagents

Favipiravir (FAV) (purity >99%) was supplied from Atabay Chemical Industry and Trade, Inc. (Istanbul, Turkey). The physicochemical properties of FAV are given in Table 1.^27^ For pH adjustment, hydrochloric acid (HCl) (0.01 N) and sodium hydroxide (NaOH) (0.1 N) were purchased from Merck. HPLC-grade reagents of methanol, sodium dihydrogen phosphate (NaH_2_PO_4_), phosphoric acid (H_3_PO_4_), hydrogen peroxide (H_2_O_2_) (30 wt %), sodium thiosulfate pentahydrate (Na_2_S_2_O_3_·5H_2_O), cobalt, zinc nitrate, and sodium sulfide were purchased from Merck. L-Cysteine was supplied from Sigma.

**Table 1:**
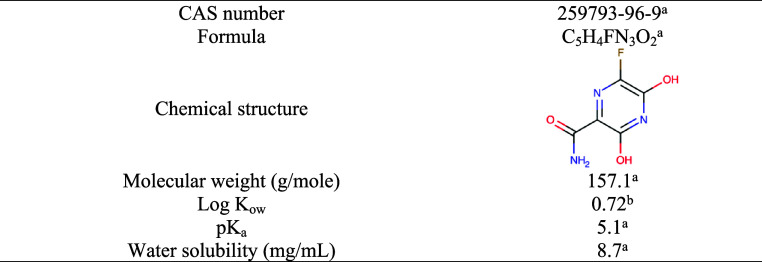
Physicochemical Properties of FAV

a Ref (30).

b Ref (4).

### 2.2 Synthesis of ZnS-L-cysteine QDs Doped with 5 wt % Cobalt (Co-Doped ZnS)

Cobalt (0.1 M) was prepared in 10 mL flasks. Five mL of the prepared solution was poured into a 100 mL flask. Zinc nitrate (0.947 g) was dissolved in 1 mL of distilled water and was poured into a balloon containing 5 mL of solution to make 0.1 M solution and made up to a volume with distilled water and deaerated for 10 min under argon gas. Next, 0.1 M L-cysteine was brought to a volume of 100 mL in a balloon and was added drop by drop to the solution. Then, 0.1 M sodium sulfide was added to the volume in a 100 mL flask and added dropwise to the solution. The resulting sediment was separated through a centrifuge and dried at 55 °C.

### 2.3 Water Samples

The distilled water was obtained from a Milli-Q-Plus system (Milli-Q Gradient A10, Millipore, Inc., USA). The WWTP effluent was collected from municipal wastewater treatment plants in Istanbul Province, Turkiye that performed the preliminary and A^2^O (anaerobic–anoxic–oxic) processes after it had been fully processed. The main characterization parameters for water samples are given in Table 2.

**Table 2:** Characterization of the Water Samples

**parameters**	**units**	**DW**	**TW**	**WWTP effluent**
pH		6.09 ± 0.37	7.12 ± 0.65	7.42 ± 0.82
conductivity	μS/cm	27.0 ± 2.54	466 ± 6.21	992 ± 9.98
TOC	mg/L	1.063 ± 0.15	1.639 ± 0.27	7.384 ± 0.87
*Anions*				
F^–^	mg/L	<0.04	<0.04	<0.04
Cl^–^		0.02 ± 0.01	59.64 ± 3.35	180.53 ± 8.54
NO_2_^–^		<0.20	<0.20	<0.20
Br^–^		<0.20	<0.20	<0.20
NO_3_^–^		<0.20	2.31 ± 0.62	5.69 ± 0.81
PO_4_^3–^		<0.40	<0.40	0.36 ± 0.03
SO_4_^2–^		n.d.	60.61 ± 7.12	94.83 ± 6.87
*Cations*				
Na^+^	mg/L	<0.20	26.7 ± 2.35	119.9 ± 8.12
NH_4_^+^		<0.125	<0.125	20.40 ± 2.76
K^+^		<0.20	3.69 ± 0.54	22.14 ± 3.05
Mg^2+^		<0.20	8.71 ± 1.23	10.51 ± 2.65
Ca^2+^		<0.20	55.07 ± 8.43	64.61 ± 9.21

### 2.4 Experimental Setup and Operation

**Figure 1:**
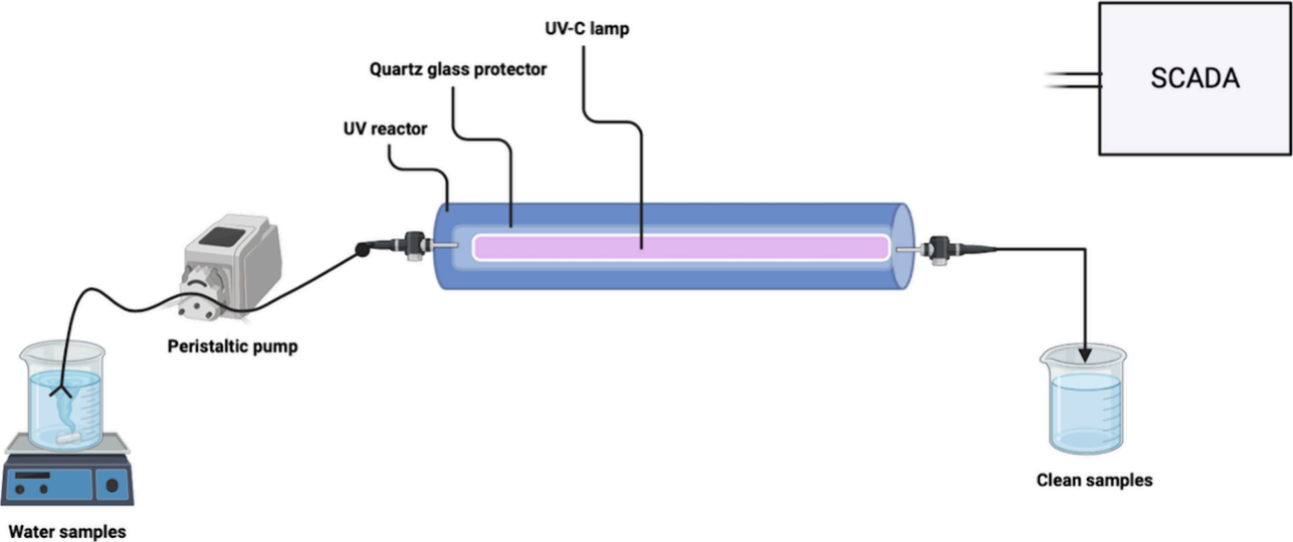
Schematic diagram of the UV system.

A schematic diagram of the UV system is presented in Figure 1. UV experiments were performed in a 21 W stainless-steel cylindrical flow-through chamber (AO Smith Purfect, ABD). The photoreactor had a working volume of 2.5 L. Quartz glass was used to protect the UV-C lamp. The samples were exposed to a UV lamp at 254 nm with a power intensity of 30,000 mWh/cm^2^. The water samples were fed into the system by using a pump at a speed of 100 rpm within 9 min. After the experiment, the water sample was withdrawn from the system after 1 min.

First, the initial antiviral drug concentration influence (ranging from 50 to 150 μg/L favipiravir in DW) on the degradation of the FAV was examined using the UV–C process. Second, to investigate the effect of water matrices on FAV removal, experiments were conducted with distilled water, tap water, surface water, and WWTP effluent. Then, the pH effect on the degradation of FAV was evaluated at 4.0, 5.0, 7.0, and 9.0. HCl (0.01 N) and NaOH (0.1 N) was used to adjust the pH of each solution. The effect of the H_2_O_2_ dosage on FAV degradation was investigated at 1, 2, 5, and 10 μM H_2_O_2_. The effect of the catalyst concentration on FAV degradation was examined at 5, 10, 20, and 50 mg/L. The photodegradation experiments were performed at constant temperature (22–25 °C). All experiments were conducted in duplicate.

The blank control group was maintained in the absence of UV radiation under dark conditions. The experimental setup and parameters for the UV light groups were identical with those of the control group. To minimize experimental error, all experiments for each group were conducted at least three times in parallel.

### 2.5 Analytical Procedure

The favipiravir concentration was analyzed using HPLC (Shimadzu Prominence 20 HPLC system) with a photodiode array (PDA) detector at 323 nm coupled with an Inertsil ODS 3 V column (5 μm, 250 × 4.6 mm). A phosphate buffer solution was prepared with NaH_2_PO_4_ and H_3_PO_4_ at pH 3.2 (90/10). The mobile phase was methanol/phosphate buffer (25/75, v.%, pH 3.2, *T* = 25 °C, RT = 6.3 min) injected at a flow rate of 1 mL/min and a 20 μL injection volume.

Anion and cation concentrations were detected using ion chromatography (IC) (Dionex ICS-3000). The pH and conductivity were measured using a pH meter (SevenCompact) and a conductivity meter (Hach HQ40d, USA), respectively. Transformation products of FAV were analyzed by using LC-MS/QTOF.

The FAV degradation rate was calculated using eq 1:
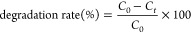
1where *C*_0_ is the initial FAV and *C_t_* is the FAV concentration in *t* time.

The pseudo-first-order (PFO) reaction model (eq 2) was used to fit the experimental data and determine the FAV degradation and mineralization rates.
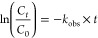
2where *k*_obs_ is the reaction rate constant and *t* is the reaction time.

Energy consumption (EC) was estimated using eq 3:^28^
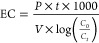
3

*P* is the rated power of the UV lamp in kW, *t* represents the reaction time in hours (h), *V* is the solution volume in liters (L), and EC is expressed in kilowatts per cubic meter (kWh/m^3^).

### 2.6 Acute Toxic Tests of Algae

An algae acute toxic test was conducted according to OECD Guideline No. 201. Twenty-five mL Erlenmeyer flasks were used for the experiment, which was carried out in an algal cabinet that was light for 24 h (21–24 °C) and randomly arranged on a shaking table. Distilled water that included different FAV contents was used as the nominal test sample, along with a fourth control vessel that solely contained medium and algae. Concentrations in each test vessel were measured using a spectrophotometer set at 540 nm at 0, 24, 48, 72, and 96 h. The temperature, light intensity, and shaking rate were measured every 24 h. Growth inhibition was calculated (eq 4):
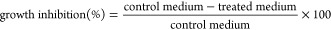
4in which the control medium means the control algal growth medium and the treated medium means treated FAV solutions.

### 2.7 Characterization of Co-Doped ZnS QDs

A diffractometer equipped with a Cu Kα source (wavelength λ = 1.541786 Å) was employed to obtain the X-ray diffraction (XRD) pattern. Fourier transform infrared spectroscopy (FTIR) was used to examine the functional groups of Co-doped ZnS QDs. The surface morphologies and elemental analysis of Co-doped ZnS QDs were determined by SEM and energy-dispersive X-ray analysis (EDX) (FEI Quanta FEG 250 model, USA). The zeta potential was measured using a Zetasizer (Malvern). Pore size distribution was determined using a dynamic light scattering instrument (Malvern, Zetasizer Pro).

### 2.8 Statistical Analysis

Statistical analysis was conducted using Microsoft Excel. The Student’s *t* test, available as a data analysis tool in Microsoft Excel, was employed to ascertain the statistical comparison, with a significance level set at *p* < 0.05.

## 3 Results and Discussion

### 3.1 Characterization of Produced Co-Doped ZnS QDs

**Figure 2:**
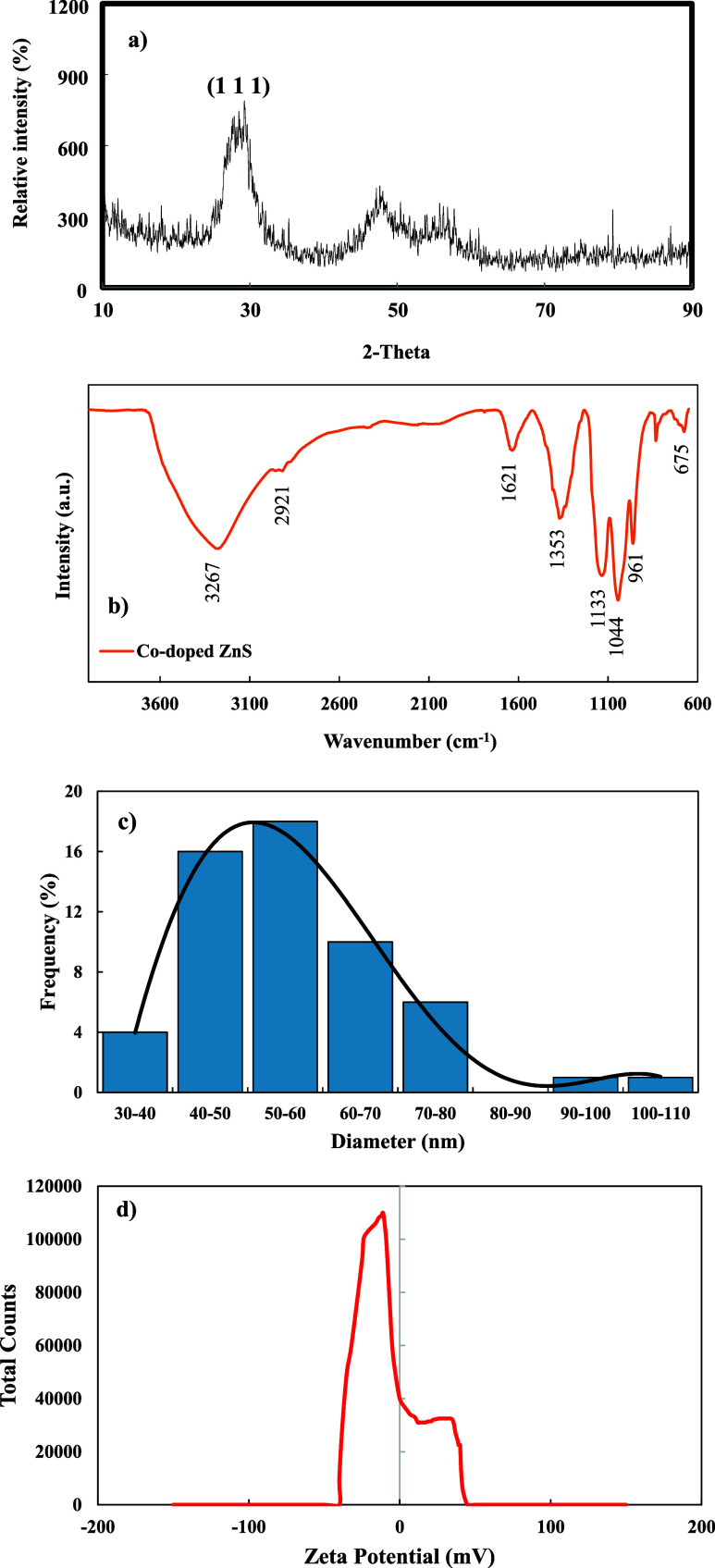
(a) XRD pattern and (b) FTIR spectrum of the Co-doped ZnS QDs. (c) Pore size distribution and (d) zeta potential of the Co-doped ZnS QDs.

To assess the phase composition and structure, the XRD pattern of Co-doped ZnS QDs was acquired and is given in Figure 2a. The diffraction peak of Co-doped ZnS QDs at 28.6° correspond to the (111) facet. All of the peaks could be identified and assigned to wurtzite ZnS (JCPDS 05-0566). Owing to the smaller dimensions of the nanoparticles under investigation, the diffraction peaks exhibit a notable degree of broadening.^29^ According to results, the Co-doped ZnS QD is crystalline and the its composition contain zinc-blend-type ZnS nanoparticles.^30^ As indicated by the XRD patterns, the dopants are effectively integrated into the framework of the ZnS structure, facilitated by their very small size. Furthermore, the doping of transition Co^2+^ metal ions into ZnS has not exerted any discernible impact on the phase. FTIR spectra of the Co-doped ZnS QDs catalyst are given in Figure 2b. The absorption band observed at 2921 cm^–1^ was associated with the stretching vibration of aliphatic CH_2_. Apart from the broad −OH band at 3000–3500 cm^–1^, the absorption band at 1621 cm^–1^ affirmed the existence of H_2_O adsorbed on the surface of nanocrystals.^31^ The absorption bands at 1400 and 1353 cm^–1^ are ascribed to the bending vibration frequency of CH_2_ and a mixed frequency involving C–O stretching and C–H bending, respectively. The absorption band observed at 961 cm^–1^ is associated with the resonance of sulfide ions’ vibrational modes in ZnS QDs. The characteristic absorption band at around 1044 cm^–1^ is attributed to the C–O stretching vibration of primary alcohols. Additionally, the absorption band at 675 cm^–1^ correlates to the metal–sulfur bond.^32,33^

**Figure 3:**
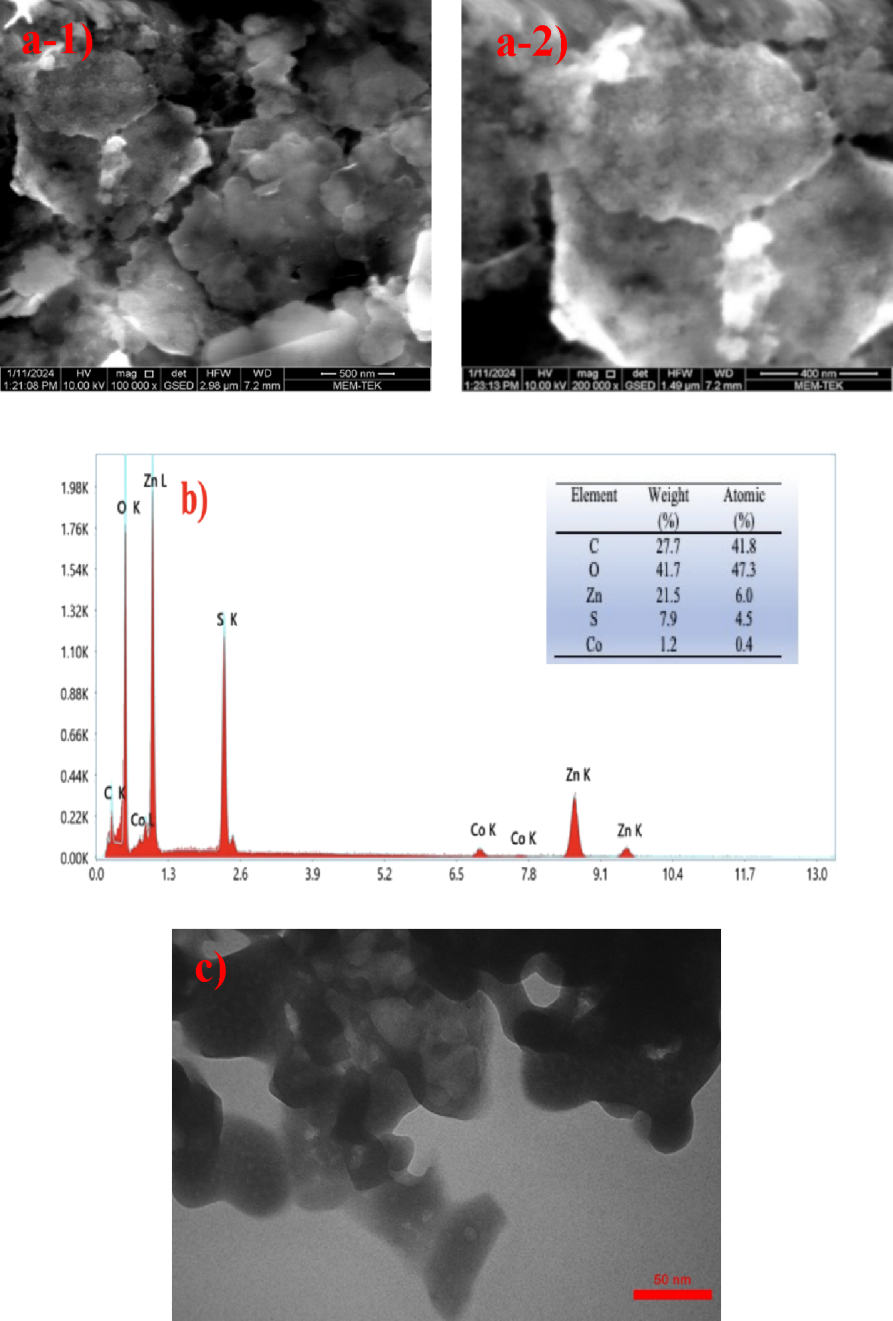
(a) SEM images, (b) EDX elemental analysis, and (c) TEM image of the Co-doped ZnS QDs.

To evaluate surface morphologies, composition, pattern of elements, and the purity of the prepared photocatalyst Co-doped ZnS QDs, SEM images, EDX analysis, and TEM images were obtained and are presented in Figure 3. As shown, the surface of Co-doped ZnS QDs appears as a rough structure. In Figure 3b, the EDX spectrum reveals prominent signals corresponding to Zn, S, and Co. The absence of any supplementary elemental peaks in the EDS spectrum signifies that the surface of the synthesized composite is devoid of impurities. This observation serves as evidence that the production process was effective in ensuring the purity of the surface. As shown in Figure 3c, the Co-doped ZnS QDs exhibited a proper spherical shape and a well-dispersed, monodisperse distribution.

The pore size and zeta potential distribution of the Co-doped ZnS QDs are given in Figures 2a and 2b, respectively. According to findings, the mean pore size was 60 nm and the average zeta potential was −7.67 mV.

### 3.2 Photolysis and Photocatalysis Experiments

#### 3.2.1 Effect of the Initial Favipiravir Concentration in the UV Process

**Figure 4:**
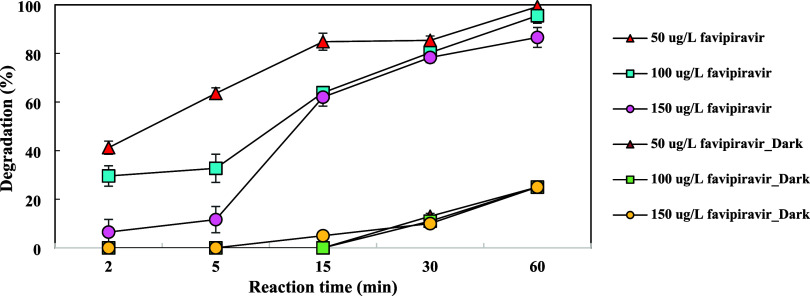
Effect of the initial favipiravir concentration on FAV degradation.

Three different FAV concentrations from 50 to 150 μg/L were prepared to investigate the effect of the initial favipiravir concentration under UV and dark conditions for 60 min. Figure 4 presents the photodegradation rate of FAV during the reaction time for initial concentrations of 50–150 μg/L. UV–vis absorption spectra of different FAV concentrations during the UV process are given in Figure S7. The results indicated that regardless of the initial FAV concentrations, residual FAV concentrations were relatively close (2–5 ppb), and the FAV photolysis rate was approximately 78–86% after 30 min of treatment. Degradation rates of FAV after 60 min were 99.2, 95.5, and 86.6% at 50, 100, and 150 μg/L initial FAV concentrations, respectively. The photolysis degradation rate was slightly decreased with an increasing initial FAV concentration. The results of the present study are consistent with those of Shokoohi et al., who investigated the photocatalytic decomposition of furfural.^34^ This finding may be explained by the fact that while the initial concentration of FAV was low, there were more than enough reactive radicals produced by UV irradiation to start the photoreactions.^35^ FAV, in itself, absorbs a portion of the irradiated rays and acts as a light filter.^34^ Under dark conditions, after 60 min, FAV was removed by 25% regardless of the initial concentration.

Also, if the overall quantities of photons were the same, a higher initial FAV concentration would have produced fewer photons per FAV molecule, resulting in a slower rate of photodegradation.^11^ The reaction rate constants (*k*) at initial FAV concentrations of 50, 100, and 150 μg/L (Figure S1) were 0.081, 0.052, and 0.033 min^–1^ at the end of the UV operation, respectively. Similar results were obtained in the study of Wang et al., in which the *k* value decreased from 0.087 to 0.024 min^–1^ with increasing initial oseltamivir phosphate concentrations from 2.6 to 53 μM in the photocatalytic reaction.^13^

#### 3.2.2 Effect of the Catalyst Concentration in the UV/Co-Doped ZnS QDs Process

**Figure 5:**
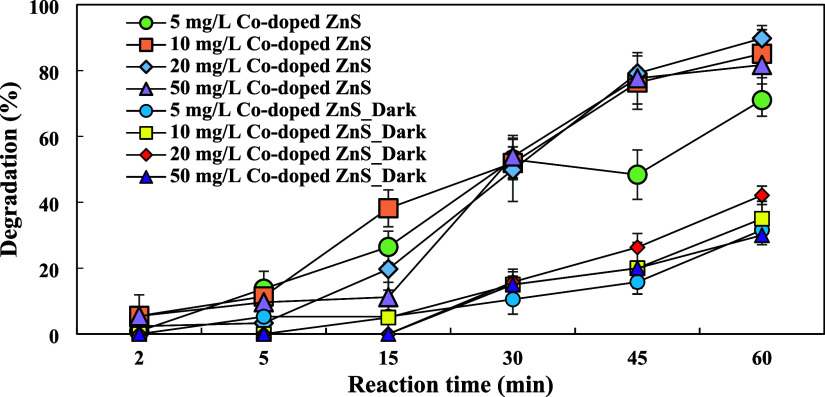
Effect of the catalyst concentration on FAV degradation.

The efficiency of the photocatalytic process is mainly linked to the exact quantity of the catalyst utilized. Employing the optimal amount of the catalyst is crucial to avoid excessive usage, which may result in particle aggregation.^19^ The effect of the Co-doped ZnS QDs concentration was examined at concentrations ranging from 5 to 50 mg/L under UV and dark conditions as shown in Figure 5. UV–vis absorption spectra of different catalyst concentrations during the UV/Co-doped ZnS QDs process are presented in Figure S14. When the catalyst concentration increased from 5 to 20 mg/L, the degradation rate of favipiravir (150 μg/L initial concentration) demonstrated a notable increase from 71.0 to 89.8% (*p* < 0.05) in 60 min. Also, the reaction rate constant as shown in Figure S2 was increased from 0.035 to 0.039 min^–1^ with an increasing catalyst concentration (from 5 to 20 mg/L). This increase is attributed to the enhanced photogeneration of active sites on the catalyst surface, leading to the formation of larger quantities of reactive oxygen species (ROS).^36^ However, above an optimum concentration (20 mg/L), the degradation rate decreased. This tendency might be explained by increased turbidity of aqueous solution, which would cause undesirable light scattering, light infiltration to the solution, and a screening effect from the excess catalyst particles.^34,37^ As a result, the optimum concentration for future experiments was determined to be 20 mg/L of the catalyst. Moreover, the photocatalytic activity of Co-doped ZnS QDs has been reported in our previous study.^23^

A similar correlation was observed under dark conditions like the UV process. As the catalyst concentration increased from 5 to 20 mg/L, the FAV removal efficiency under dark conditions increased from 31.6 to 42.1% in 60 min. However, when the catalyst concentration reached 50 mg/L, the FAV removal efficiency decreased to 30%.

#### 3.2.3 Effect of the H_2_O_2_ Dosage in the UV/H_2_O_2_ Process

**Figure 6:**
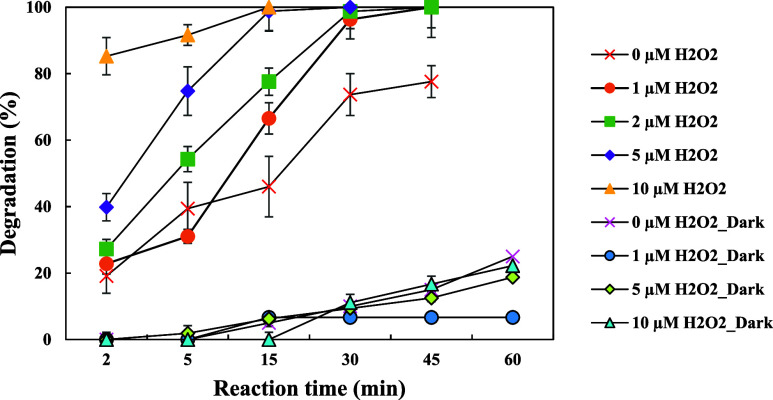
Effect of the H_2_O_2_ dosage on FAV degradation in the UV/H_2_O_2_ process.

Since H_2_O_2_ is the primary source of hydroxyl radicals (•OH) produced by H_2_O_2_-based processes, the H_2_O_2_ concentration is an extremely important component.^38^ Therefore, the effect of the H_2_O_2_ dosage ranging from 1 to 10 μM on the FAV (150 μg/L initial concentration) degradation rate in the UV/H_2_O_2_ process was examined in this study under UV and dark conditions and the results are given in Figure 6. UV–vis absorption spectra of different H_2_O_2_ dosages during the UV/H_2_O_2_ process are presented in Figure S11. The FAV degradation rate was increased with adding H_2_O_2_ from 1 to 10 μM to the process, and >99% FAV degradation was accomplished in 15 min of reaction time in the presence of 10 μM H_2_O_2_. This is due to the fact that the •OH can be generated through the UV/H_2_O_2_ process as shown in eq 5. Higher H_2_O_2_ concentrations cause more UV to be absorbed, which increases the rate at which •OH are generated. Consequently, FAV decomposes more quickly with greater •OH concentrations. As a result, when H_2_O_2_ in solution is exposed to UV light, •OH are created, which enhances the degradation of FAV.^39^

Degradation rates of FAV after 15 min were 46.2, 66.6, 77.6, 98.8, and >99% at 0, 1, 2, 5, and 10 μM H_2_O_2_ concentrations, respectively. Initially, FAV was degraded quickly at a fixed dose of H_2_O_2_ and then FAV concentration gradually decreased (*p* < 0.05). This behavior is explained by the large amount of unselective radicals (•OH) produced in the UV/H_2_O_2_ system, which break down micropollutants in water efficiently.^40^ The FAV degradation rate may be decreased due to competition from significant numbers of intermediates produced during the UV/H_2_O_2_ treatment for UV light and •OH radicals, which might occur as the reaction duration increases.^41^ Similar results were obtained in the study of Liu et al.^39^ Also, the degradation of FAV through advanced oxidation processes involves complex chemical reactions. Higher concentrations of H_2_O_2_ can accelerate these reactions by providing more reactants and increasing the likelihood of collision between reactant molecules. This acceleration in the reaction kinetics leads to a faster degradation rate of FAV.
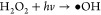
5

The *k* values of FAV degradation as shown in Figure S3 increased with an increasing H_2_O_2_ dosage. The *k* values of FAV after 5 min were 0.100, 0.074, 0.157, 0.275, and 0.496 min^–1^ at 0, 1, 2, 5, and 10 μM H_2_O_2_ concentrations, respectively.

Additionally, under dark conditions, the FAV removal efficiency increased with higher concentrations of H_2_O_2_. Removal efficiencies of 6.6, 8.8, 18.7, and 22.2% were achieved for H_2_O_2_ concentrations of 1, 2, 5, and 10 μM, respectively.

#### 3.2.4 Effect of Water Matrices in UV, UV/H_2_O_2_, and UV/Co-Doped ZnS QDs Processes

**Figure 7:**
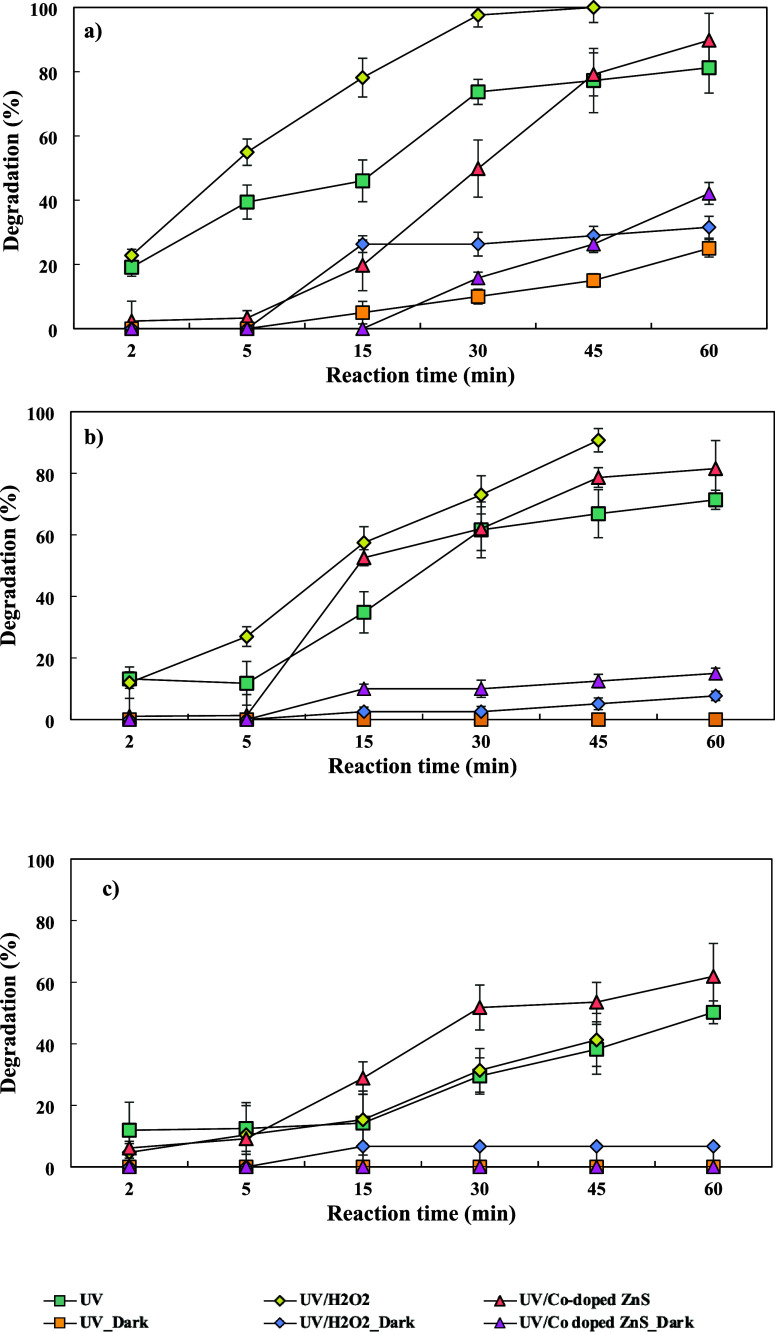
Effect of water matrices in UV, UV/H_2_O_2_, and UV/Co-doped ZnS QDs processes on the FAV degradation rate: (a) DW, (b) TW, and (c) WWTP (favipiravir = 150 μg/L, H_2_O_2_ = 1 μM, and Co-doped ZnS QDs = 20 mg/L).

The impact of water matrices on the UV, UV/H_2_O_2_, and UV/Co-doped ZnS QDs processes was examined under UV and dark conditions since water components like organic anions, cations, and natural organic matter influence the degradation of pollutants by scavenging radicals during AOP processes.^42,43^ Generally, as the complexity of the water matrix increases, the effectiveness of UV-based advanced oxidation processes (AOPs) in removing micropollutants such as antibiotics decreases.^44^Figure 7 shows the performance of the UV, UV/H_2_O_2_, and UV/Co-doped ZnS QDs processes during the reaction time in the treatment of distilled water, a wastewater treatment plant effluent, and a tap water sample spiked with 150 μg/L FAV. UV–vis absorption spectra of FAV degradation for each water matrix are presented in Figures S8, S10, and S13 for UV, UV/H_2_O_2_, and UV/Co-doped ZnS QDs processes, respectively. After 60 min, degradations of FAV with spiked DW, TW, and WWTP effluents were 81.2, 71.4, and 50.2% in the UV process, respectively. After 45 min, FAV degradations were 100, 90.7, and 41.2% in order of DW, TW, and WWTP effluents in the UV/H_2_O_2_ process, respectively. In the UV/Co-doped ZnS QDs process, degradation rates of favipiravir in DW, TW, and WWTP effluent after 60 min were found to be 89.8, 81.5, and 61.9%, respectively. As expected, the lower degradation rate in WWTPE in UV, UV/H_2_O_2_, and UV/Co-doped ZnS QDs processes compared with DW and TW was due to higher levels of initial organic matter present in the WWTP effluent. While dissolved organic matter (DOM) in the water matrix is a significant scavenger of free radicals, its consumption of chemical oxidants and UV irradiation is limited.^45^ Also, results showed that there were significant differences in the degradation rate of favipiravir for each aqueous matrix in the presence of a Co-doped ZnS QDs catalyst. According to a study on FAV removal using electrochemical oxidation, the FAV removal efficiency in pure water was 98.6%, whereas FAV removal efficiency in wastewater was 87.7%, which is consistent with the results of a present study.^7^ Anions like Cl^–^, NO_2_^–^, and NO_3_^–^ can cause photoshielding effects. Therefore, a longer irradiation time can be needed during the treatment of FAV in different water matrices.^46^

The *k* values of DW, TW, and WWTP effluent as shown in Figure S4 after 30 min of the UV process were 0.045, 0.032, and 0.012 min^–1^, while *k* values after 30 min were 0.110, 0.044, and 0.013 min^–1^ in UV/H_2_O_2_, respectively. In the UV/Co-doped ZnS QDs process, the *k* values for DW, TW, and WWTP were found to be 0.023, 0.032, and 0.024 min^–1^, respectively. *k* values decreased with increasing anions and dissolved organic matter (DOM) concentrations (Table 1). A similar trend was obtained in the study of Paniagua et al.^47^ This can be explained by the fact that high concentrations of water components can either compete with FAV for reactive species or eliminate UV light to reduce UV photolysis of FAV.^48^ The other reason is that the higher DOC levels in the water sample might react with •OH radicals to produce more radical scavengers.^49^ Also, Tran et al. stated that the presence of water matrix components substantially altered the degradation pathways of photocatalysis, thereby influencing the degradation efficiency of the photocatalysis process.^50^

Under dark conditions, three distinct processes were evaluated, each with three different water matrices. For each process, the removal efficiencies varied, depending on both the process and the water matrix. In the UV process under dark conditions, the removal efficiencies were 25, 0, and 0% for DW, TW, and WWTP, respectively. In UV/H_2_O_2_ under dark conditions, the removal efficiencies were 6.6, 31.5, and 7.7% for the same water matrices. Finally, in the UV/Co-doped ZnS QDs process under dark conditions, the removal efficiencies were 42.1, 15, and 0% for DW, TW, and WWTP, respectively. These results suggest that both the type of process and the characteristics of the water matrix play significant roles in determining the FAV removal efficiency under dark conditions.

#### 3.2.5 Effect of pH in UV, UV/H_2_O_2_, and UV/Co-Doped ZnS QDs Processes

**Figure 8:**
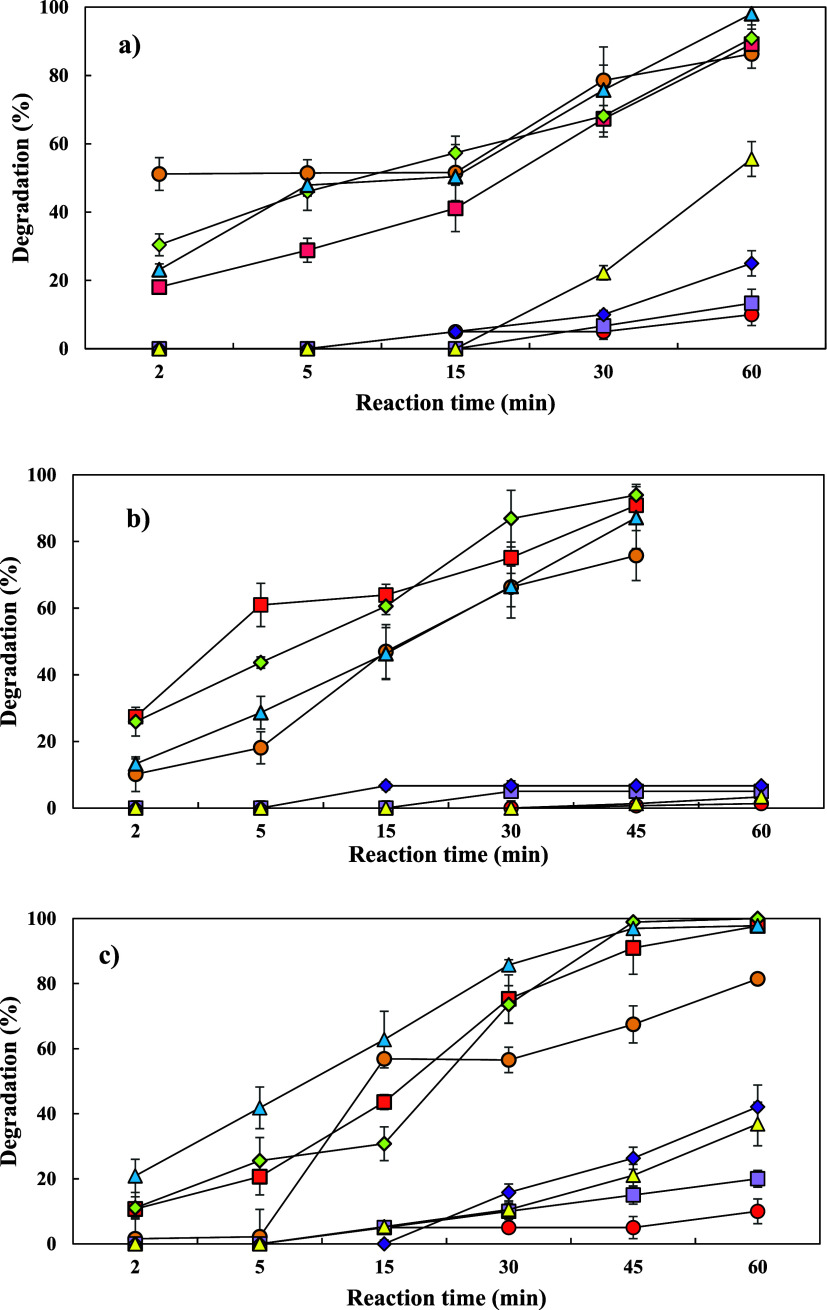
Effect of pH on the FAV degradation rate in (a) UV, (b) UV/H_2_O_2_, and (c) UV/Co-doped ZnS QDs processes (favipiravir = 150 μg/L, H_2_O_2_ = 1 μM, and Co-doped ZnS QDs = 20 mg/L).

The effect of pH on FAV degradation (distilled water spiked with 150 μg/L FAV) using UV, UV/H_2_O_2_, and UV/Co-doped ZnS QDs processes was studied at pH 4.0, 5.0, 7.0, and 9.0 under UV and dark conditions. Figure 8 shows the effect of pH on degradation of FAV by UV, UV/H_2_O_2_, and UV/Co-doped ZnS QDs processes and dark conditions during the reaction time. The effect of pH on the PFO rate constant is given in Figure S5. UV spectra of FAV degradation for different pH values are given in Figures S9, S12, and S15 for UV, UV/H_2_O_2_, and UV/Co-doped ZnS QDs processes, respectively. The pH had a considerable effect on the photolysis rate of FAV for both UV and UV/H_2_O_2_ processes. The degradation rate increased with increasing pH values (*p* < 0.05) in the UV process. After 60 min of UV irradiation, 86.3, 89.1, 90.9, and 98.0% amounts of FAV were degraded at pH 4.0, 5.0, 7.0, and 9.0, respectively. The reaction rate constants (*k*) of FAV at four different pH values (4.0, 5.0, 7.0, and 9.0) were 0.047, 0.037, 0.037, and 0.048 min^–1^ after 45 min, respectively. The *k* value increased from pH 4 to pH 9, and the photolysis rate of FAV at pH 9.0 was faster than that at pH 4.0. Also, FAV removal efficiencies were 10, 13.3, 25, and 55.5% for pH 4.0, 5.0, 7.0, and 9.0 under dark conditions, respectively. Kiyanmehr et al. reported that the FAV removal efficiency using the VUV/O_3_ process in terms of measured *k*_obs_ at pH 8.0 was almost 1.4 times that at pH 4.0.^8^ The photolysis degradation rate of FAV was the fastest under alkaline conditions, followed by neutral and acidic conditions (alkaline > neutral > acidic). This result can be explained by considering the dissociation of FAV. Taking into account the p*K*_a_ of FAV (5.1), it is disintegrated into its ionic form in an aqueous solution with pH 7.0 and 9.0 and has higher reactivity than the undissociated forms, resulting in a higher degradation rate than under acidic conditions (pH 4.0 and pH 5.0).^8^ At pH 7.0 and 9.0, FAV is nearly completely dissociated (p*K*_a_ = 5.1), and its electron cloud density is higher in the dissociated state, which makes it easier for it to transfer electrons and accelerates the reaction.^51^

In the UV/H_2_O_2_ process, a maximum degradation rate was observed at neutral pH, especially pH 7.0, and the lowest degradation rates were observed at pH 4.0 and pH 9.0. FAV degradation rates after 45 min were 75.8, 90.9, 93.9, and 87.2% at pH 4.0, 5.0, 7.0, and 9.0, respectively. Under dark conditions, 1.3, 5.0, 6.7, and 3.3% amounts of FAV were degraded at pH 4.0, 5.0, 7.0, and 9.0, respectively. In addition, *k* values for 45 min of the UV/H_2_O_2_ process were 0.032, 0.053, 0.062, and 0.046 min^–1^ at pH 4.0, 5.0, 7.0, and 9.0, respectively. According to eq 6, the lower degradation rate at pH 4.0 can be explained by the additional H^+^ ions acting as OH radical scavengers.^49^
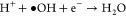
6

The following factors can be the cause of the reduced FAV degradation under alkaline conditions. First, hydroxyl radicals readily combine with OH^–^ in alkaline solutions to create O^–^, which has a low oxidative capacity (eq 7), lowering the concentration of hydroxyl radicals.
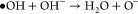
7

Second, H_2_O_2_ deprotonation is producing hydroperoxide anions, which, in accordance with eq 8, react with a nondissociated H_2_O_2_ molecule to generate dioxygen and water.^49,52^
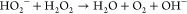
8

Third, there is a considerable rise in the H_2_O_2_ self-decomposition rate (eq 9) when the pH value increases.^39^
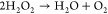
9

Photocatalytic degradation predominantly occurs on the surface of the photocatalyst. Therefore, the pH of the solution plays a fundamental role in the efficiency of the photocatalysis process, influencing the surface charge of the photocatalyst.^53^ Moreover, the pH has the potential to influence interactions among reactant species, the catalyst surface, and the solvent. It can also impact the aggregation of Co-doped ZnS QDs, the types of radicals formed, and the generation of transformation products (TPs).^37^ In the UV/Co-doped ZnS QDs process, FAV degradations obtained were 81.4, 97.7, 100, and 97.8% at pH 4.0, 5.0, 7.0, and 9.0, respectively. Under dark conditions, FAV removal efficiencies were 10.0, 20.0, 42.1, and 36.8% for pH 4.0, 5.0, 7.0, and 9.0, respectively. After 45 min, *k* values were found 0.025, 0.051, 0.100, and 0.056 min^–1^ at pH 4.0, 5.0, 7.0, and 9.0, respectively. The surface charge of Co-doped ZnS QDs in the basic medium is negative, so it is thought to prevent the formation of hydroxyl radicals in the basic medium due to the repulsive force between the Co-doped ZnS QDs and OH^–^ ions.

### 3.3 Comparison of UV, UV/H_2_O_2_, UV/Co-Doped ZnS QDs, and UV/H_2_O_2_/Co-Doped ZnS QDs Processes

**Figure 9:**
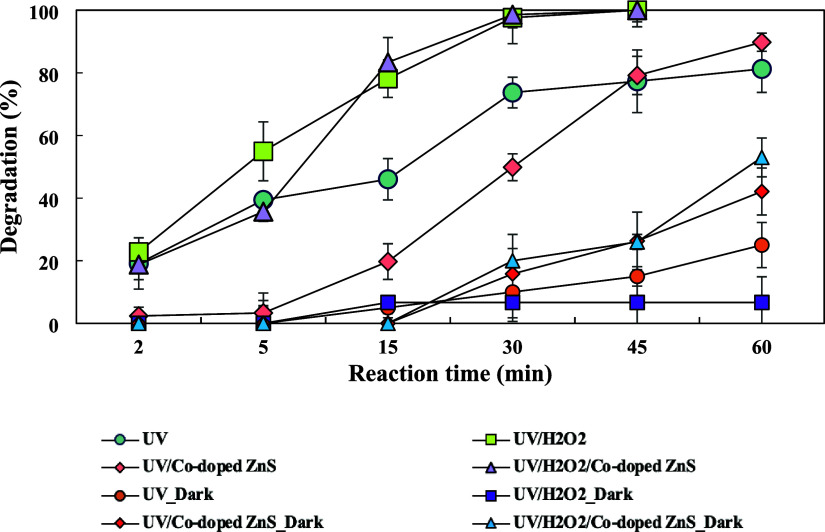
Comparison of UV, UV/H_2_O_2_, UV/Co-doped ZnS QDs, and UV/H_2_O_2_/Co-doped ZnS QDs processes for the FAV degradation rate (favipiravir = 150 μg/L, H_2_O_2_ = 1 μM, and Co-doped ZnS QDs = 20 mg/L).

Figure 9 shows favipiravir degradation performance for UV, UV/H_2_O_2_, UV/Co-doped ZnS QDs, and UV/H_2_O_2_/Co-doped ZnS QDs processes under UV and dark conditions. The degradation rate of favipiravir (distilled water spiked with 150 μg/L for an initial FAV concentration and pH 6.09) in the UV/H_2_O_2_/Co-doped ZnS QDs process was significantly higher than those in the UV and UV/Co-doped ZnS QDs process. After 45 min of experiments, FAV degradations were found to be 77.3, 100, 89.8, and 100% in UV, UV/H_2_O_2_, UV/Co-doped ZnS QDs, and UV/H_2_O_2_/Co-doped ZnS QDs processes, respectively. FAV removal efficiencies were 25, 6.7, 42.1, and 53.0% for UV, UV/H_2_O_2_, UV/Co-doped ZnS QDs, and UV/H_2_O_2_/Co-doped ZnS QDs processes under dark conditions, respectively. Also, the favipiravir degradation rate by UV/H_2_O_2_ was 96.4%, while a 98.6% favipiravir degradation rate was obtained using the UV/H_2_O_2_/Co-doped ZnS QDs process. Results showed that the Co-doped ZnS QDs as a catalyst improved the efficiency of the UV process and increased the favipiravir degradation rate from 81.2 to 89.8% at the end of 60 min. *k* values for the initial 30 min in UV, UV/H_2_O_2_, UV/Co-doped ZnS QDs, and UV/H_2_O_2_/Co-doped ZnS QDs processes as shown in Figure S6 were 0.045, 0.110, 0.023, and 0.141 min^–1^, respectively.

### 3.4 Mineralization and Energy Consumption during FAV Degradation

**Figure 10:**
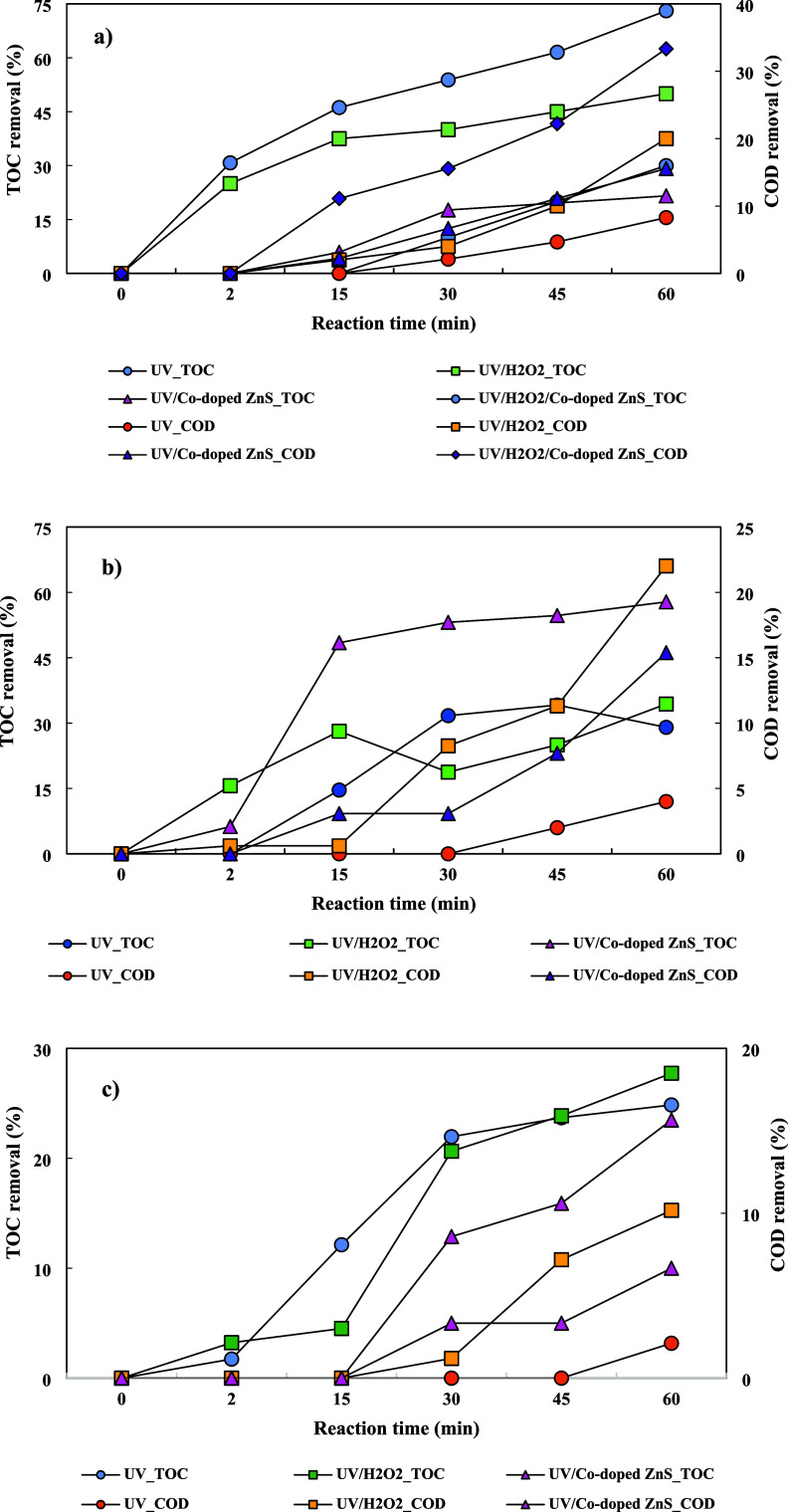
TOC and COD removal efficiency for different matrices and different UV processes: (a) DW, (b) TW, and (c) WWTP.

To evaluate the mineralization capability of the UV, UV/H_2_O_2_, and UV/Co-doped ZnS QDs processes, COD and TOC removal efficiencies were determined for different water matrices as given in Figure 10. TOC removal efficiencies were 30.0, 50.0, 21.5, and 73.0% for UV, UV/H_2_O_2_, UV/Co-doped ZnS QDs, and UV/H_2_O_2_/Co-doped ZnS QDs processes using DW in 60 min, respectively. COD removal efficiencies were 8.2, 20.0, 15.5, and 33.3% for UV, UV/H_2_O_2_, UV/Co-doped ZnS QDs, and UV/H_2_O_2_/Co-doped ZnS QDs processes with DW in 60 min, respectively. TOC removal efficiencies were found to be 39.0, 34.3, and 57.8% for UV, UV/H_2_O_2_, and UV/Co-doped ZnS QDs processes using TW in 60 min, respectively. COD was removed by UV, UV/H_2_O_2_, and UV/Co-doped ZnS QDs processes up to 4.0, 22.0, and 15.3% for using TW in 60 min, respectively. TOC removal efficiencies of the UV, UV/H_2_O_2_, and UV/Co-doped ZnS QDs processes were 24.8, 27.7, and 23.4%, respectively, when treating the WWTP effluent for 60 min. COD removal efficiencies were 2.1, 10.1, and 6.6% for UV, UV/H_2_O_2_, and UV/Co-doped ZnS QDs processes using the WWTP effluent in 60 min. Mineralization was incomplete for all evaluated matrices. Moreover, the lower mineralization observed in all processes for the WWTP effluent compared to DW and TW was attributed to the higher initial organic matter content in the WWTP effluent.^47^

**Figure 11:**
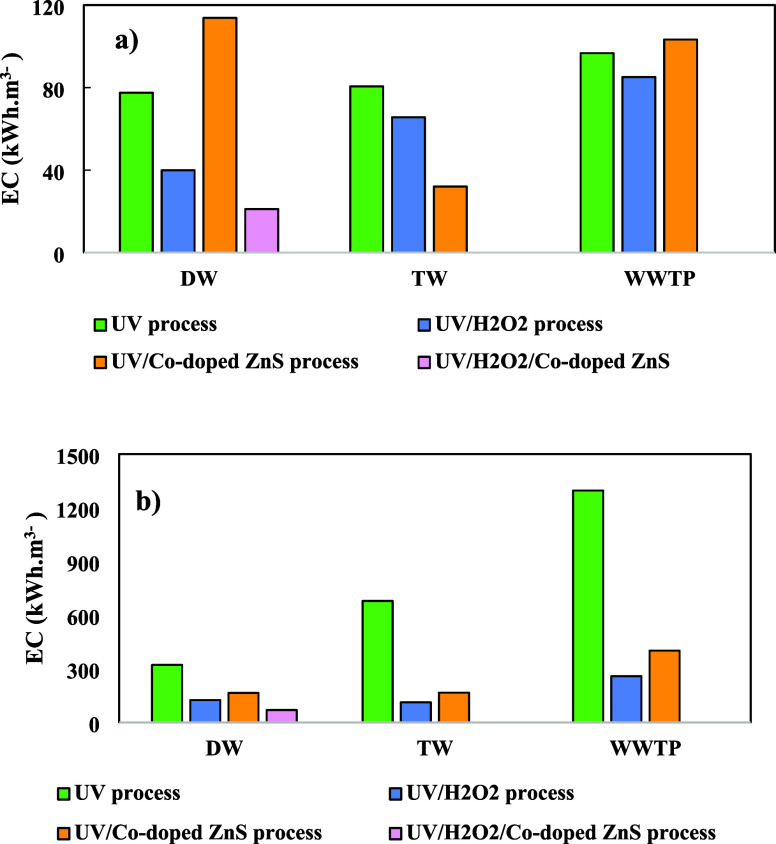
Energy consumption during the mineralization of FAV in different processes as a function of (a) TOC and (b) COD.

Energy consumption is a critical parameter in evaluating the feasibility and sustainability of treatment processes.^54−58^ The energy consumption as a function of COD and TOC removal during FAV degradation in 60 min for three different UV processes is shown in Figure 11. As observed, the UV/Co-doped ZnS QDs process mostly had the lowest energy consumption, while the UV process had the highest for different water matrices. Energy consumption depends on the initial organic matter concentration and increases with an increasing organic matter concentration (from DW to the WWTP effluent).^59,60^ Compared to energy consumption as a function of COD and TOC removal in the UV process, the UV/Co-doped ZnS QDs process mostly reduced energy consumption. Samarghandi et al. reported that the inclusion of GAC particle electrodes decreases the system’s energy consumption by approximately 41%, which is consistent with the findings of this study.^61^

### 3.5 Proposed Degradation Pathways of FAV in UV and UV/Co-Doped ZnS QDs Processes

**Figure 12:**
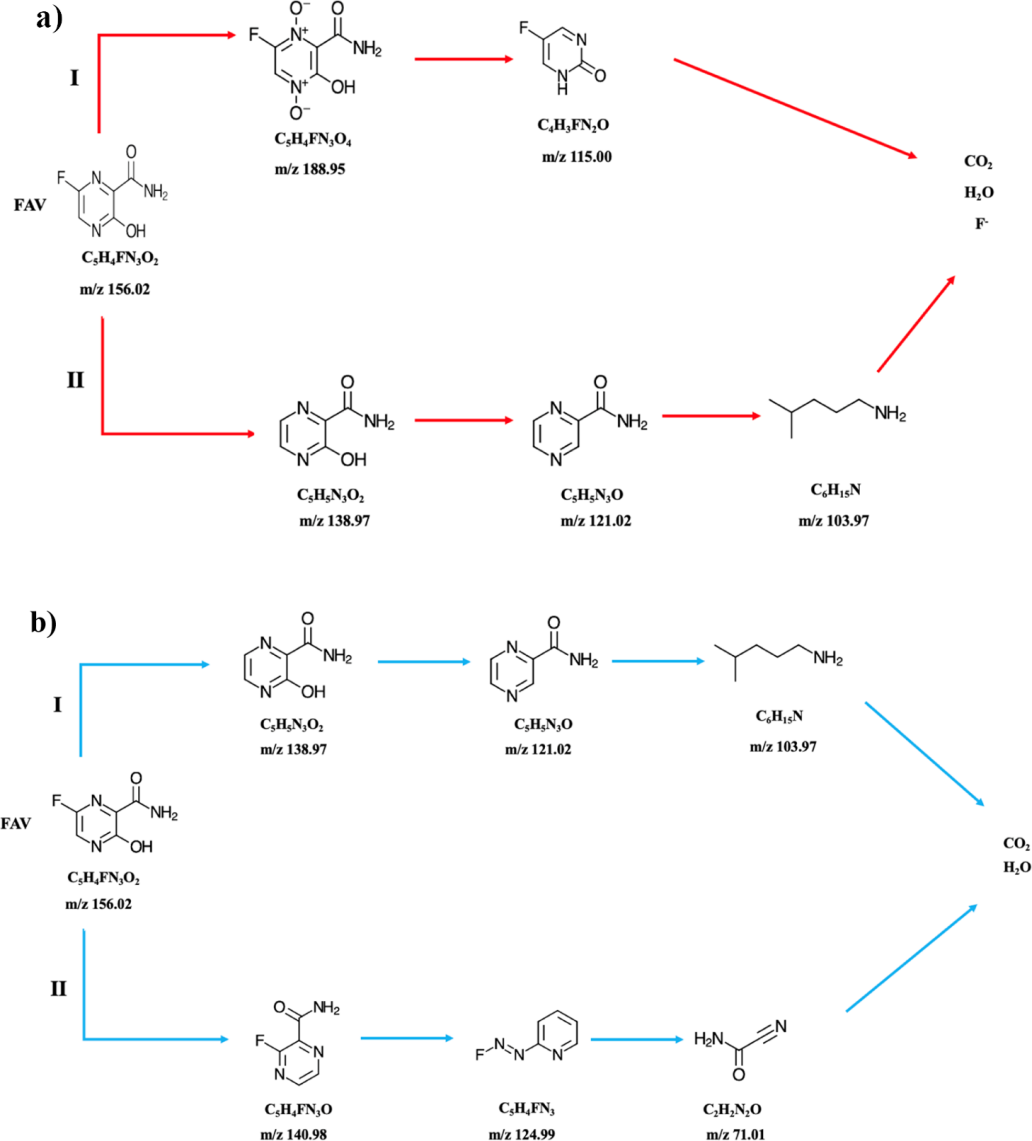
Proposed degradation pathways of FAV in (a) UV process and (b) UV/Co-doped ZnS QDs process.

The degradation intermediates of FAV during the UV and UV/Co-doped ZnS QDs processes were analyzed using LC-MS/QTOF under optimal experimental conditions over reaction times. Transformation products (TPs) were identified based on their mass-to-charge (*m*/*z*) values, as detailed in Tables S1 and S2. LC-MS/QTOF spectra of FAV degradation products are presented in Figure S16. Utilizing the detected intermediates, the degradation pathway of FAV was proposed and is illustrated in Figure 12. The overall degradation pathway primarily involves ring-opening mechanisms, including defluorination, deamidation, dehydroxylation, denitrogenation, and breakdown of the pyrazine heterocycle.^62^ Pathways I and II in UV and UV/Co-doped ZnS QDs processes were generated in the initial reaction steps through the dehydroxylation and defluorination of FAV, primarily via interactions with generated radicals. Prolonged reaction times led to denitrogenation and benzene ring cleavage, resulting in the formation of short-chain organic acids. Notably, as the reaction progressed and additional HO• radicals were produced, the intensity of aromatic compounds diminished gradually, with some intermediates persisting in solution, while others completely disappeared.

### 3.6 Ecotoxicity Assessment of Treated FAV Solutions

**Figure 13:**
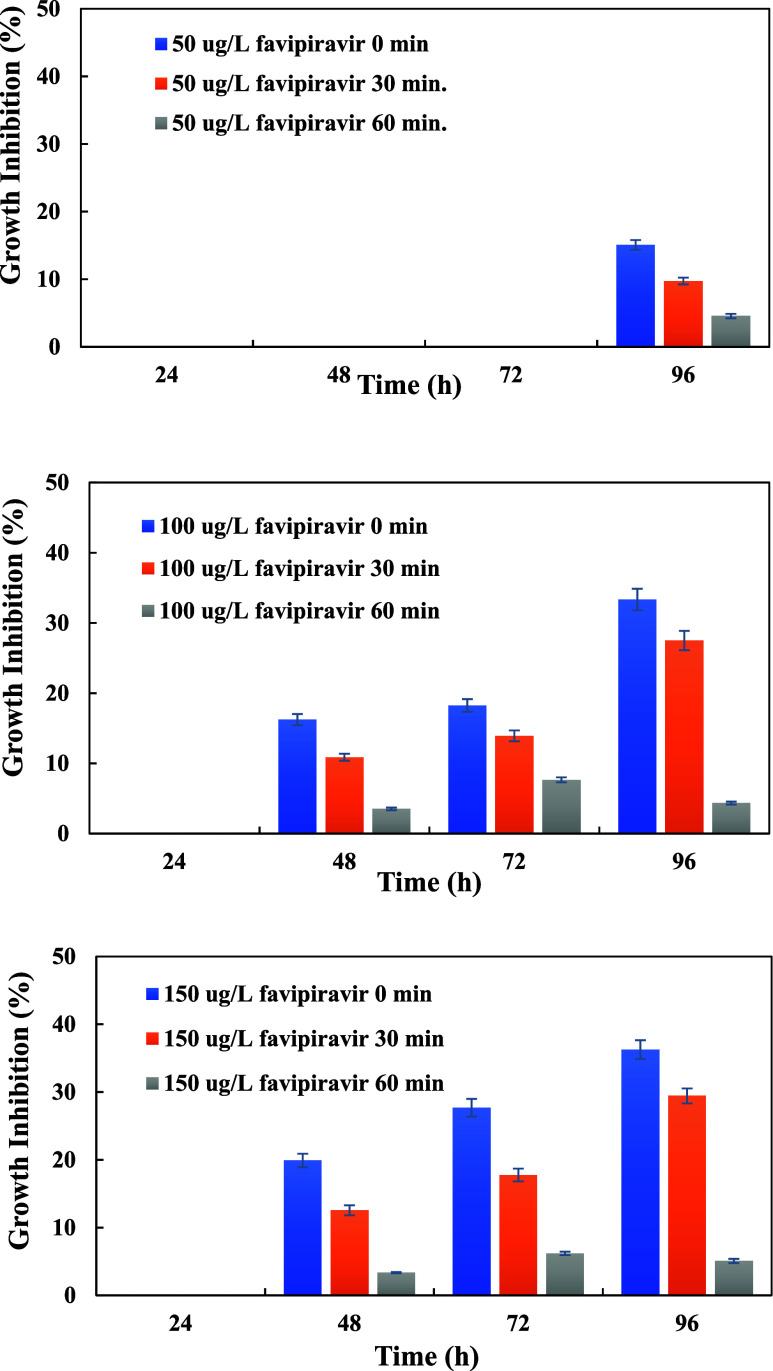
Ecotoxicity assessment of FAV using a treated solution.

Since antivirals may be harmful and according to (Q)SAR, modeling and toxicity data rank eighth among the most dangerous therapeutic classes to aquatic living beings such as daphnia, algae, and fish, ecotoxicity assessments for treated solutions that included different FAV concentrations were conducted using algae.^9^ The toxicity effect of FAV on algal growth during 96 h is shown in Figure 13. Regardless of the initial FAV concentration, FAV has no detrimental effect on algal growth in 24 h. Growth inhibition rates for 50, 100, and 150 μg/L initial FAV concentrations were detected to be 0, 16.2, and 19.9% at 48 h, respectively (*p* < 0.05). Growth inhibition rates for 50, 100, and 150 μg/L FAV in distilled water were 15.1, 33.3, and 36.3% at 96 h, respectively. While the initial FAV concentration was 50 μg/L, it inhibited algal growth only at the end of 96 h. It showed an inhibition effect on algal growth at the end of 48 h for 100 and 150 μg/L FAV initial concentrations. After a 30 min UV treatment, the growth inhibition rate decreased from a 33.3 to a 27.5% growth inhibition at 96 h for the initial concentration of 100 μg/L FAV while it was reduced from 36.2 to 29.4% for the initial concentration of 150 μg/L FAV. Regardless of the concentration, the inhibition effect of the FAV solution on algae was less than 1% after UV treatment for 60 min. The algal growth inhibition following UV treatments exhibited a more pronounced effect at the onset of degradation, gradually diminishing as the degradation period extended. That is, the treatment duration of AOPs should be carefully determined to ensure the safety of the ecological environment. Also, the toxicity was eliminated as the UV reaction time increased. Meng et al. stated that the functional structure of the FAV intermediate products was gradually broken down as the treatment period increased, resulting in the elimination of toxicity.^7^ In the study of Kuroda et al., it has been found that the removal of favipiravir by conventional systems will be low, so it will be found in the secondary effluents and therefore in the environment, and the ecotoxicological risk will be high in river waters.^4^

### 3.7 Comparison with literature data

Table 3 shows a comparison of the efficiency of the processes utilized in this study with the results obtained from studies on the removal of FAV using various treatment processes. Compared to the literature, the removal of antiviral drugs through the photocatalytic process facilitated by the addition of UV/Co-doped ZnS QDs demonstrates higher efficacy (favipiravir removal of 89.8% for the photocatalytic process). Therefore, the UV/Co-doped ZnS QDs process can be an effective option for degrading antiviral drugs in aqueous matrices.

**Table 3:** Comparison of Process Performance of FAV Removal with Other Processes

**antiviral drug**	**process**	**water matrix**	**concentration**	**process conditions**	**degradation rate (%)**	**references**
FAV	electro-Fenton process	aqueous solutions	32.5 mg/L	pH = 2.7, Fe^2+^ concentration = 47.49 mg/L, reaction time = 92.5 min, and current density = 226.25 mA/cm^2^	97.8%	(10)
FAV	MBR	synthetic wastewater	10.7 mg/L	membrane content: 16 wt % poly(ether imide) (PEI), 4 wt % polyethylene glycol (PEG), 77.5 wt % *N*-methyl-2 pyrrolidone (NMP), 0.5 wt % ZIF-8, 1.5 wt % MoS_2_, 2.5 wt % h-BN SRT = 35 days, and HRT = 8 h	87.5%	(63)
FAV	vacuum UV/ozonation (VUV/O_3_)	tap water	10 mg/L	pH = 8, O_3_ = 0.35 mg/min, and reaction time = 2 min	100%	(8)
		real wastewater effluent		pH = 7.71, O_3_ = 0.35 mg/min	100%	
FAV	electrochemical oxidation			Ti/TiO_2_-nanotube arrays (NTA)/Ti_4_O_7_, reaction time = 120 min	100%	(7)
				Ti_4_O_7_, reaction time = 120 min	98.7%	
FAV	photocatalysis	distilled water	500 ng/mL	Ag/Ag_(30%)_-SG-TiO_2_-rGO photocatalyst concentration = 0.087 g, pH = 8, and volume = 25 mL	100%	(64)
		tap water			92.7%	
		sewage treatment plant effluent			75.3%	
FAV	UV	distilled water	150 μg/L	reaction time = 45 min	77.3%	present study
	UV/H_2_O_2_			H_2_O_2_ = 1 μM, reaction time = 45 min	100%	
	UV/Co-doped ZnS QDs			Co-doped ZnS QDs = 20 mg/L, reaction time = 45 min	89.8%	
	UV/H_2_O_2_/Co-doped ZnS QDs			H_2_O_2_ = 1 μM, Co-doped ZnS QDs = 20 mg/L, and reaction time = 45 min	100%	

## 4 Conclusions

This work highlights the significance of investigating UV, UV/H_2_O_2_, and UV/Co-doped ZnS QDs processes degradation of favipiravir in different matrices to find out how the matrix composition, pH, H_2_O_2_ dosage, initial favipiravir concentration, and catalyst concentration impact the removal rate, degradation kinetics, and toxicity. The favipiravir degradation rate and removal efficiency increased with an increase in pH due to changes in favipiravir ionization in the UV process. In contrast, the maximum FAV degradation rate was obtained at neutral pH and a lower FAV degradation rate was observed under both acidic and alkaline conditions in UV/H_2_O_2_ and UV/Co-doped ZnS QDs processes. The degradation rate of FAV was enhanced with an increasing H_2_O_2_ dosage in the UV/H_2_O_2_ process. Additionally, the aqueous matrix compositions, particularly the natural organic matter, had a significant impact on the rates and efficiencies of the favipiravir degradation. The initial favipiravir concentration has a relatively minor effect on the degradation kinetics and rate of favipiravir. The FAV degradation increased with addition of the catalyst. The degradation pathway of FAV in the developed process was assessed, revealing HO• as the predominant reactive species. The favipiravir inhibition effect on algal growth started after 48 h for 100 and 150 μg/L, while it emerged after 96 h for 50 μg/L. The FAV inhibition effect on algal growth was decreased with UV treatment time. Overall, the UV/Co-doped ZnS QDs process was helpful for the favipiravir degradation in all tested aqueous matrices; however, a preliminary treatment step is advised for the removal of organic matter from the environment.

## Supporting Information Available

Effect of the initial favipiravir concentration on the PFO reaction rate constant; effect of the catalyst concentration on the PFO reaction rate constant; effect of the H_2_O_2_ dosage on the PFO reaction rate constant the in UV/H_2_O_2_ proces; effect of water matrices in UV, UV/H_2_O_2_, and UV/Co-doped ZnS QDs processes on the PFO reaction rate constant; effect of pH on the PFO reaction rate constant; PFO rate constant for comparison of UV, UV/H_2_O_2_, UV/Co-doped ZnS QDs, and UV/H_2_O_2_/Co-doped ZnS QDs processes; UV–vis absorption spectra of different FAV concentrations during the UV process; UV–vis absorption spectra of different water matrices during the UV process; UV–vis absorption spectra of different pH during the UV process; UV–vis absorption spectra of different water matrices during the UV/H_2_O_2_ process; UV–vis absorption spectra of different H_2_O_2_ dosages during the UV/H_2_O_2_ process; UV–vis absorption spectra of different pH dosages during the UV/H_2_O_2_ process; UV–vis absorption spectra of different water matrices during the UV/Co-doped ZnS QDs process; UV–vis absorption spectra of different catalyst concentrations during the UV/Co-doped ZnS QDs process; UV–vis absorption spectra of different pH dosages during the UV/Co-doped ZnS QDs process; identified transformation products in FAV degradation by the UV process; identified transformation products in FAV degradation by the UV/Co-doped ZnS QDs process; LC-MS/QTOF spectra at negative mode ([M-H]^−^) of FAV degradation products (PDF)
