# Supplementary material for: Degradation of Antiviral Drug Favipiravir Using UV, UV/H2O2, and Photocatalysis with Co-Doped ZnS Quantum Dots: Operational Parameters, Kinetic Studies, and Toxicity Assessment
Source: Langmuir. 2025 Mar 5;41(10):6528–43. doi: 10.1021/acs.langmuir.4c03639 (PMC11924239; doi:10.1021/acs.langmuir.4c03639)
Supplement: Supplementary file 1 — la4c03639_si_001.pdf [file la4c03639_si_001.pdf]

Supporting Information

Degradation of antiviral drug favipiravir using UV, UV/H_2O_2 and photocatalysis with Co-doped ZnS quantum dots: Operational parameters, kinetic studies and toxicity assessment

Bahriye Eryildiz-Yesir [a,b], Hale Ozgun [a,b], Mustafa Evren Ersahin [a,b], Hamid Reza Rajabi [c], Vahid Vatanpour [a,d], Ismail Koyuncu [a,b,*]

[a] Istanbul Technical University, Environmental Engineering Department, Maslak, 34469, Istanbul, Turkey

[b] National Research Center on Membrane Technologies, Istanbul Technical University, Maslak, 34469, Istanbul, Turkey

[c] Chemistry Department, Yasouj University, Yasouj, 75918-74831, Iran

[d] Department of Applied Chemistry, Faculty of Chemistry, Kharazmi University, Tehran,15719-14911, Iran

*** Corresponding Author:** koyuncu@itu.edu.tr

Contents

Fig. S1. Effect of initial favipiravir concentration on PFO reaction rate constant…………..S-4

Fig. S2. Effect of catalyst concentration on PFO reaction rate constant…………………….S-4

Fig. S3. Effect of H_2O_2 dosage on PFO reaction rate constant in UV/H_2O_2 proces…………S-5

Fig. S4. Effect of water matrixes in UV, UV/H_2O_2 and UV/Co-doped ZnS QDs processes on PFO reaction rate constant: a) DW b)TW c)WWTP (Favipiravir= 150 µg/L, H_2O_2= 1 µM, Co-doped ZnS= 20 mg/L)………………………………………………………………………S-6

Fig. S5. Effect of pH on PFO reaction rate constant a) pH 4 b) pH 5 c) pH 7 d) pH 9 (Favipiravir= 150 µg/L, H_2O_2= 1 µM, Co-doped ZnS= 20 mg/L)…………………………..S-7

Fig. S6. PFO rate constant for comparison of UV, UV/H_2O_2, UV/Co-doped ZnS QDs and UV/H_2O_2/Co-doped ZnS QDs process (Favipiravir= 150 µg/L, H_2O_2= 1 µM, Co-doped ZnS= 20 mg/L)………………………………………………………………………………..S-8

Fig. S7. UV-Vis absorption spectra of different FAV concentrations during UV process a) 50 µg/L FAV b) 100 µg/L FAV c) 150 µg/L FAV………………………………………………S-9

Fig. S8. UV-Vis absorption spectra of different water matrixes during UV process a) DW; b) TW; c) WWTP………………………………………………………………………...S-10

Fig. S9. UV-Vis absorption spectra of different pH during UV process a) pH 4 b) pH 5 c) pH 7 d) pH 9…………………………………………………………………………………S-11

Fig. S10. UV-Vis absorption spectra of different water matrixes during UV/H_2O_2 process a) DW b) TW c) WWTP………………………………………………………………S-12

Fig. S11. UV-Vis absorption spectra of different H_2O_2 dosage during UV/H_2O_2 process: a) 0 µM H_2O_2; b) 1 µM H_2O_2 c) 2 µM H_2O_2 d) 5 µM H_2O_2 e) 10 µM H_2O_2…………………...S-13

Fig. S12. UV-Vis absorption spectra of different pH dosage during UV/H_2O_2 process: a) pH 4 b) pH 5 c) pH 7 d) pH 9……………………………………………………………S-15

Fig. S13. UV-Vis absorption spectra of different water matrixes during UV/Co-doped ZnS QDs process a) DW b) TW c) WWTP…….………………………………………………S-16

Fig. S14. UV-Vis absorption spectra of different catalyst concentrations during UV/Co-doped ZnS QDs process a) 5 mg/L b) 10 mg/L c) 20 mg/L d) 50 mg/L…………………………..S-17

Fig. S15. UV-Vis absorption spectra of different pH dosage during UV/Co-doped ZnS QDs process : a) pH 4 b) pH 5 c) pH 7 d) pH 9……………………………...………………..S-18

Fig. S16. LC-MS/QTOF spectra, at negative mode ([M-H]$^-$) of FAV degradation products……………………………………………………………………………………S-22

Table S1. The identified transformation products in FAV degradation by the UV process..S-20

Table S2. The identified transformation products in FAV degradation by the UV/Co-doped ZnS QDs process……………………………………………………………………...S-21

Number of pages: 23

Number of figures: 16

Number of tables: 2

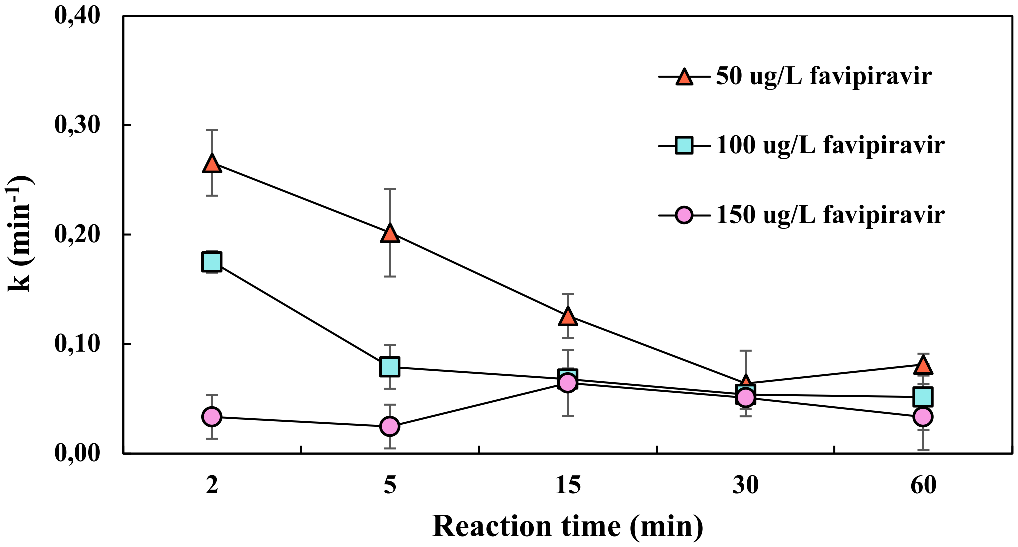

Fig. S1. Effect of initial favipiravir concentration on PFO reaction rate constant.

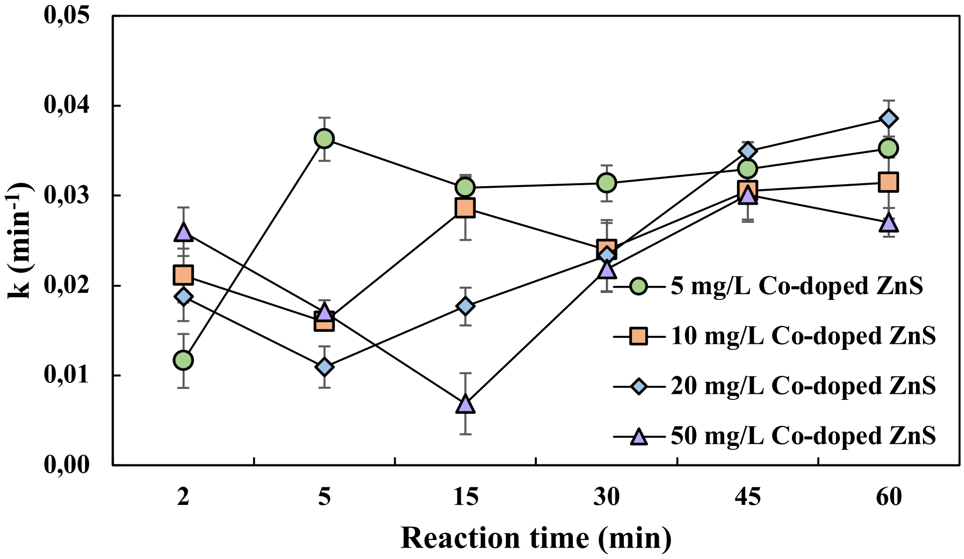

Fig. S2. Effect of catalyst concentration on PFO reaction rate constant.

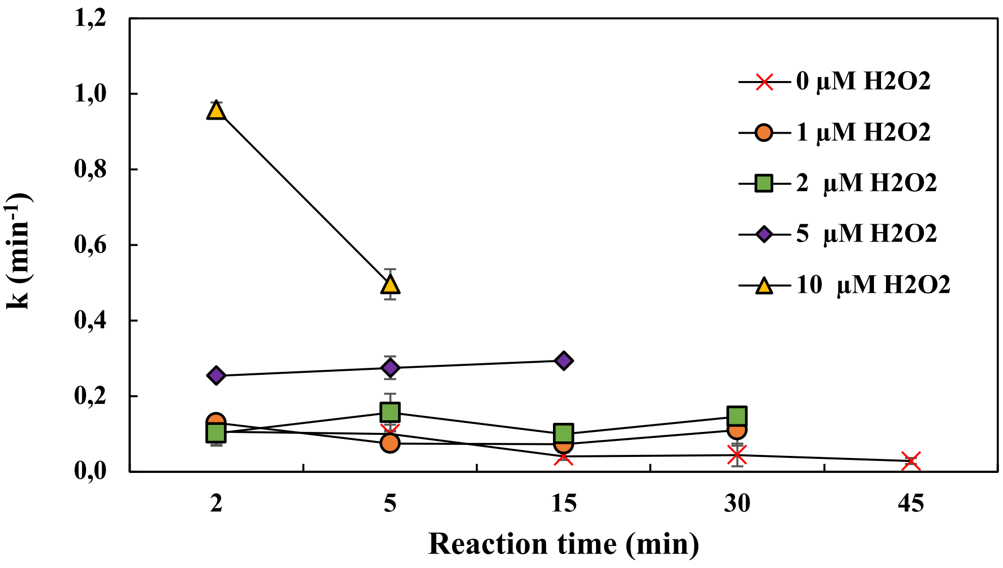

Fig. S3. Effect of H_2O_2 dosage on PFO reaction rate constant in UV/H_2O_2 proces.

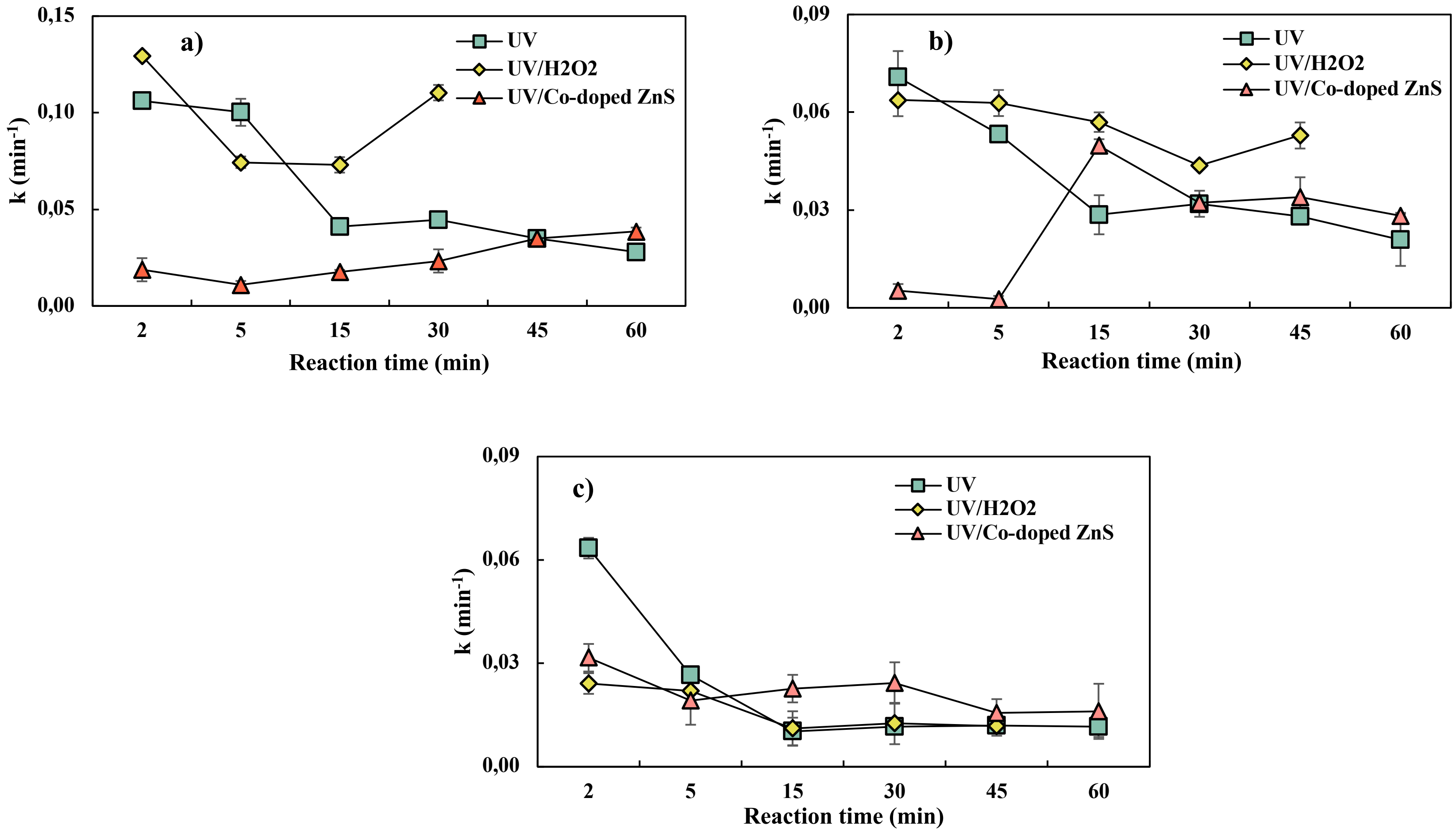

Fig. S4. Effect of water matrixes in UV, UV/H_2O_2 and UV/Co-doped ZnS QDs processes on PFO reaction rate constant: a) DW b)TW c)WWTP

(Favipiravir= 150 μg/L, H_2O_2= 1 μM, Co-doped ZnS QDs= 20 mg/L).

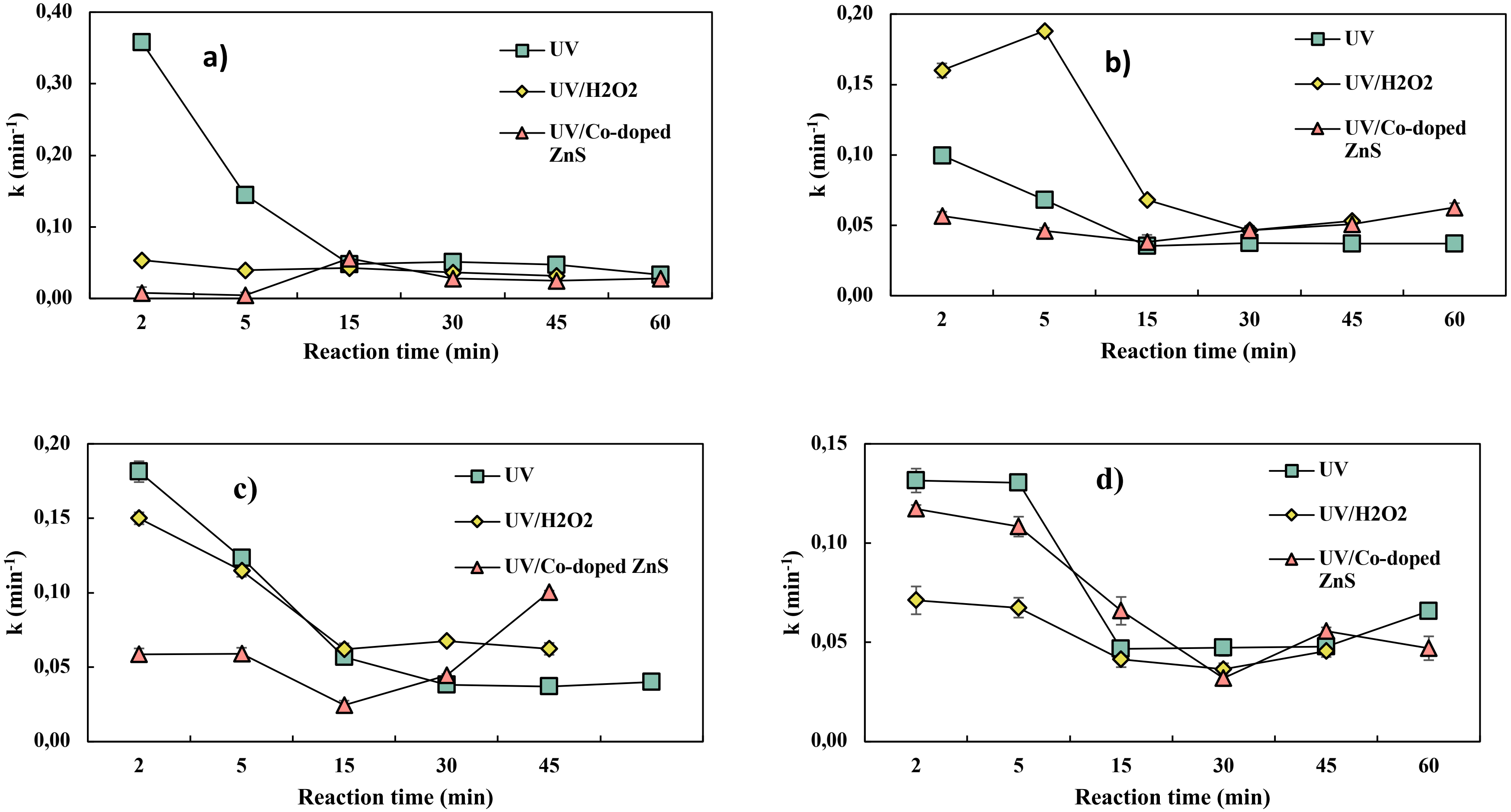

Fig. S5. Effect of pH on PFO reaction rate constant a) pH 4 b) pH 5 c) pH 7 d) pH 9 (Favipiravir= 150 µg/L, H_2O_2= 1 µM, Co-doped ZnS QDs= 20 mg/L).

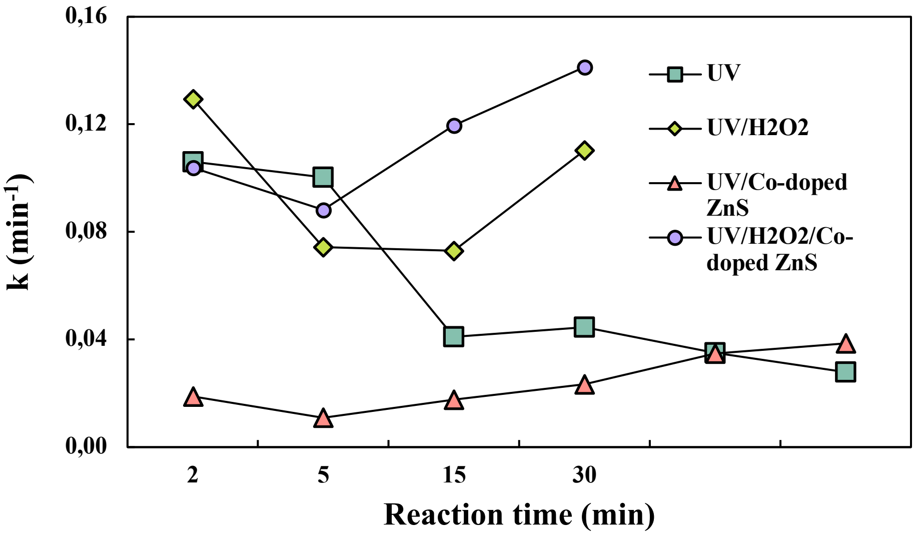

Fig. S6. PFO rate constant for comparison of UV, UV/H_2O_2, UV/Co-doped ZnS QDs and UV/H_2O_2/Co-doped ZnS QDs process (Favipiravir= 150 μg/L, H_2O_2= 1 μM, Co-doped ZnS QDs= 20 mg/L).

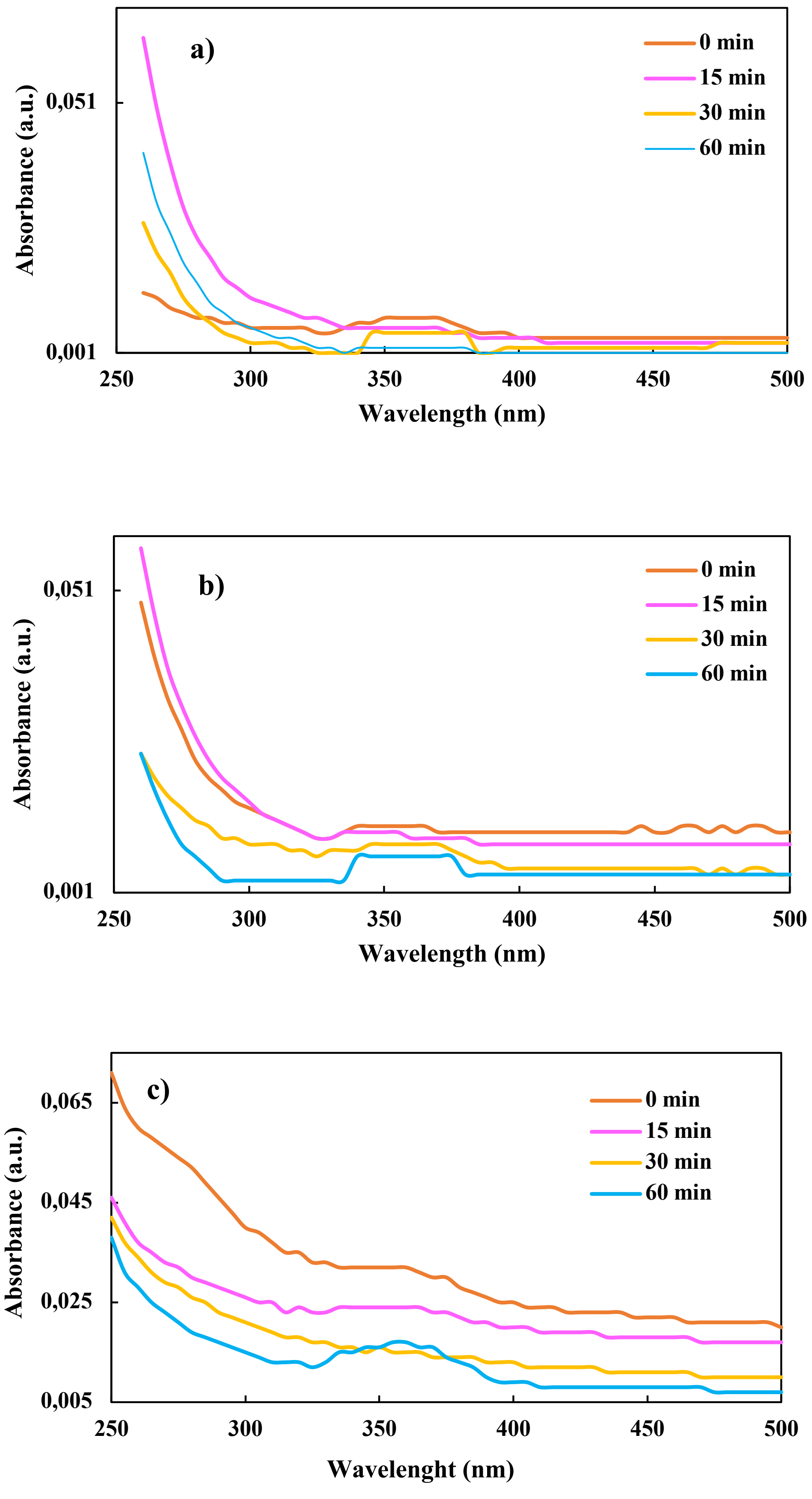

Fig. S7. UV-Vis absorption spectra of different FAV concentrations during UV process a) 50 µg/L FAV b) 100 µg/L FAV c) 150 µg/L FAV.

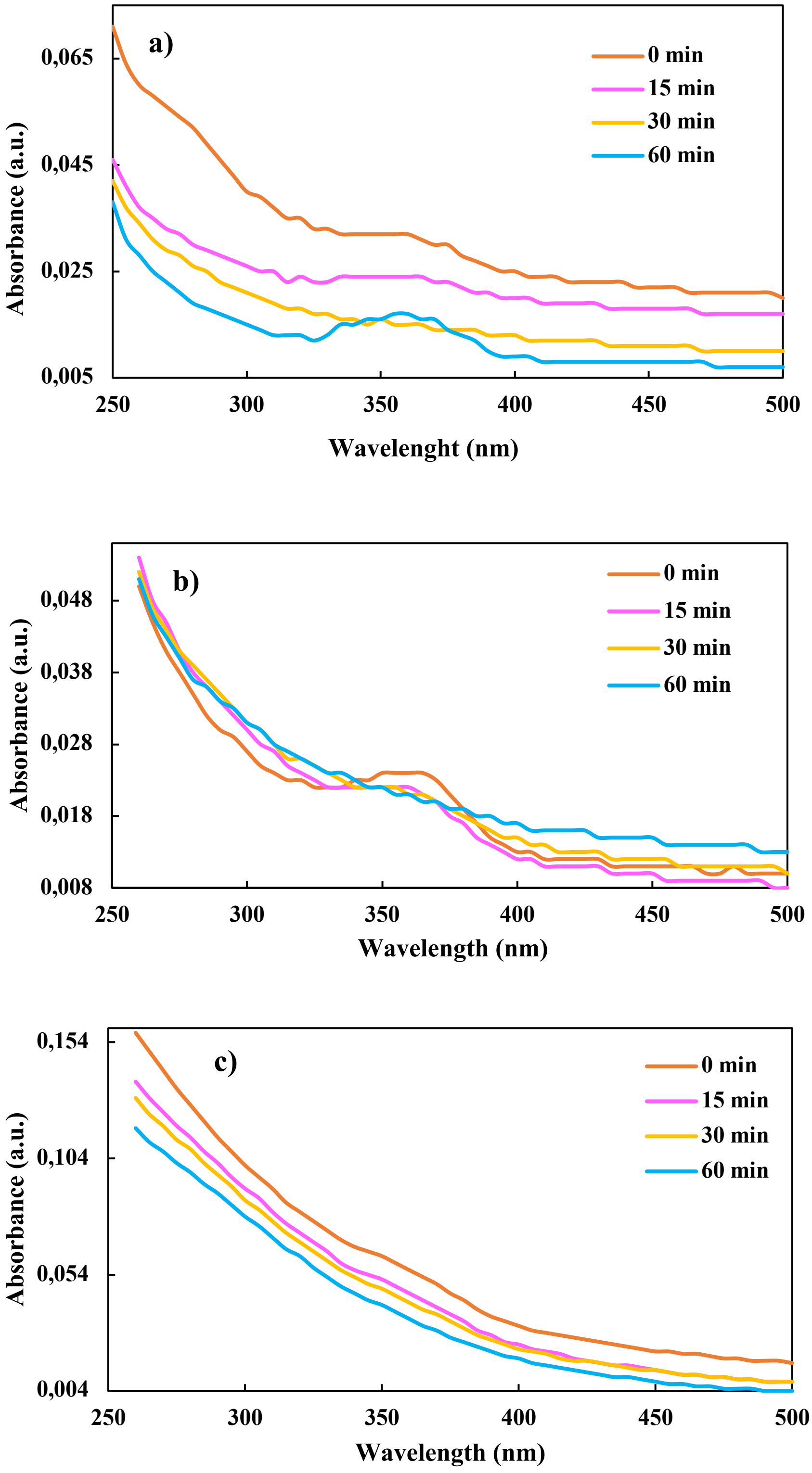

Fig. S8. UV-Vis absorption spectra of different water matrixes during UV process a) DW; b) TW; c) WWTP.

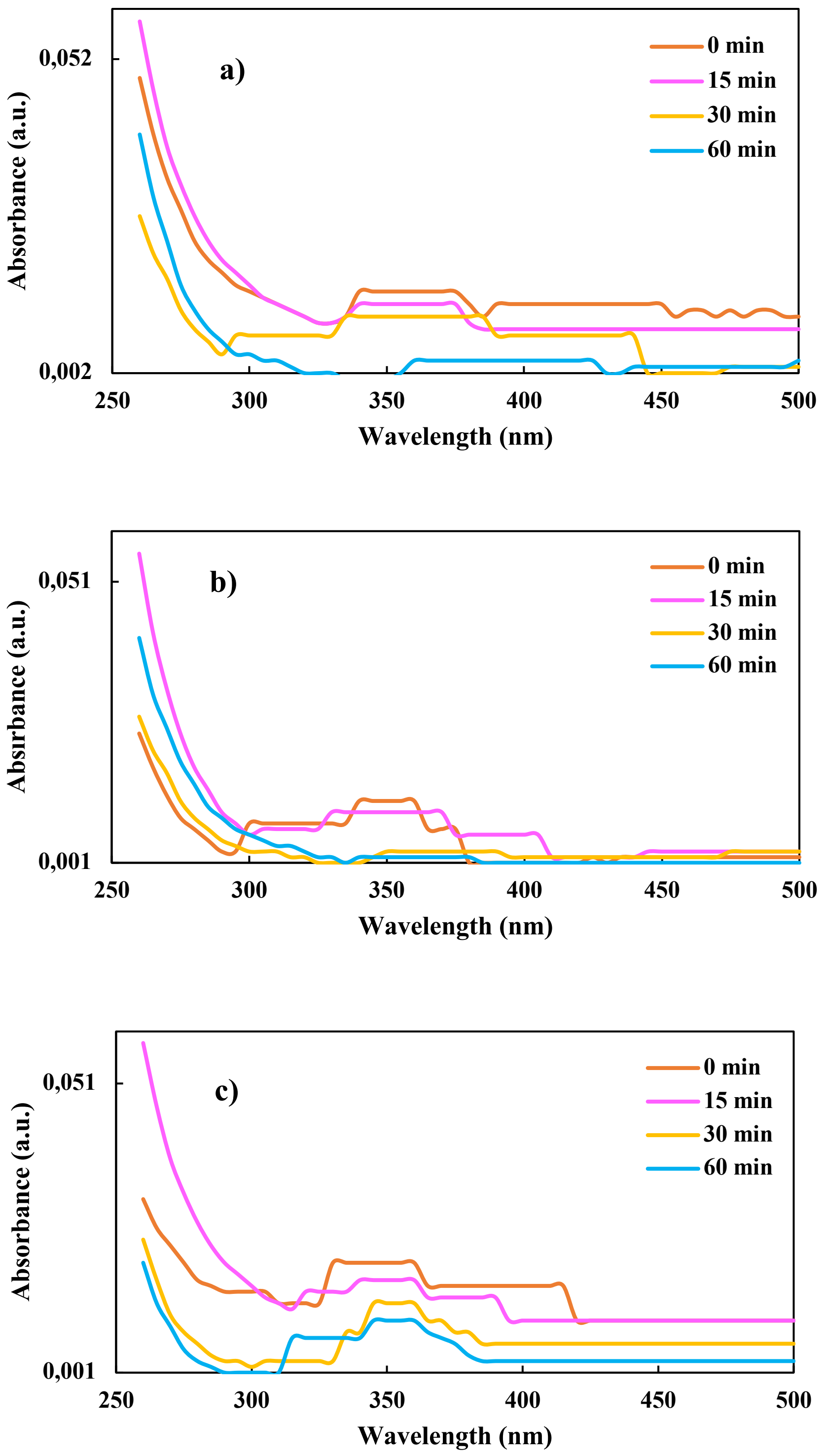

Fig. S9. UV-Vis absorption spectra of different pH during UV process a) pH 4 b) pH 5 c) pH 7 d) pH 9.

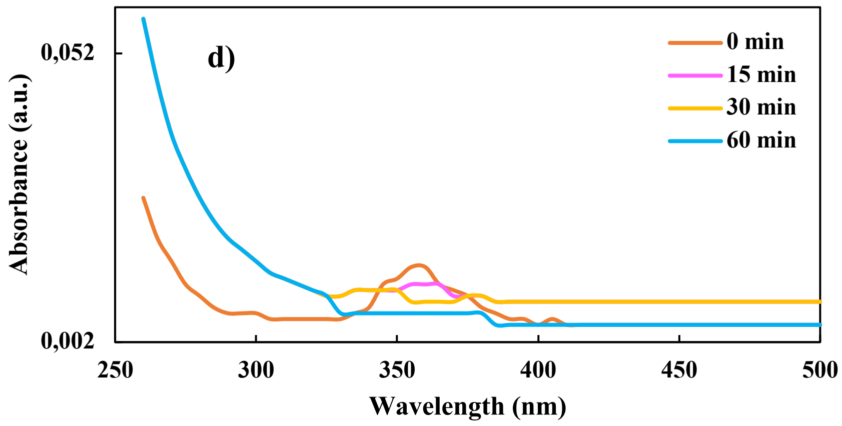

Fig. S9. *(continued).*

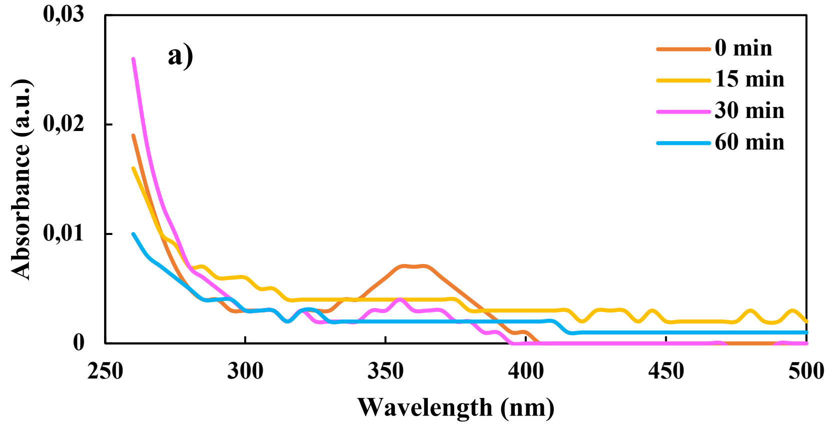

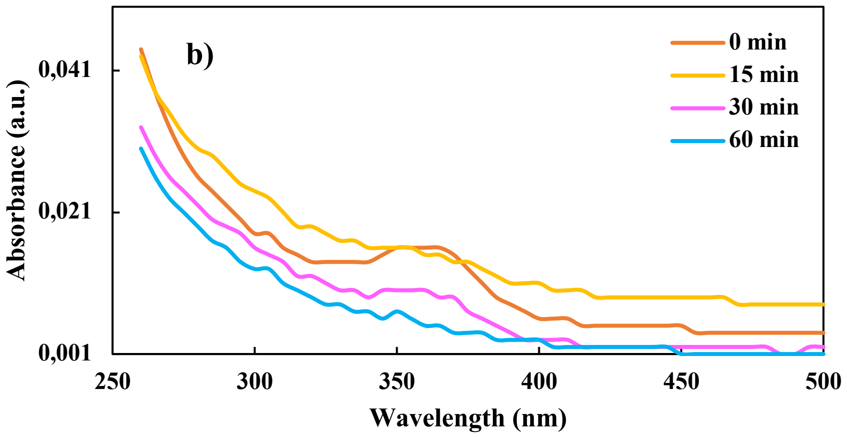

Fig. S10. UV-Vis absorption spectra of different water matrixes during UV/H_2O_2 process a) DW b) TW c) WWTP.

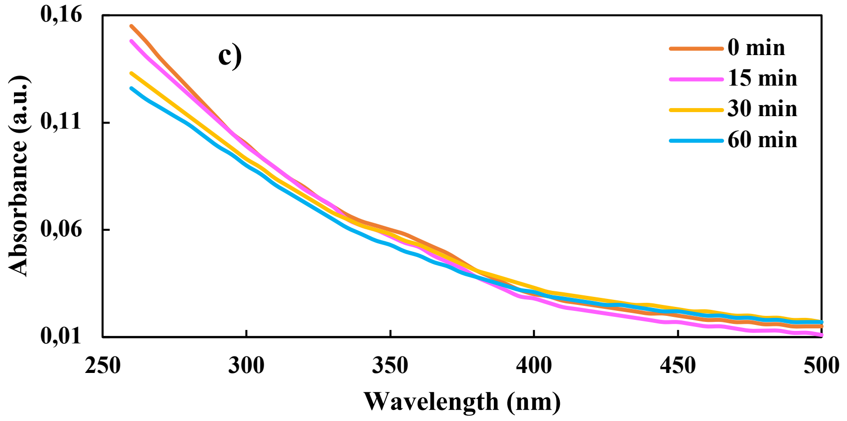

Fig. S10. *(continued)*.

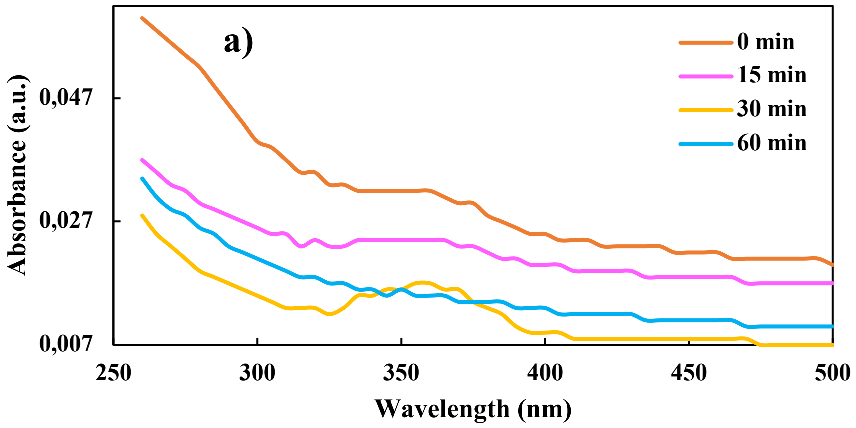

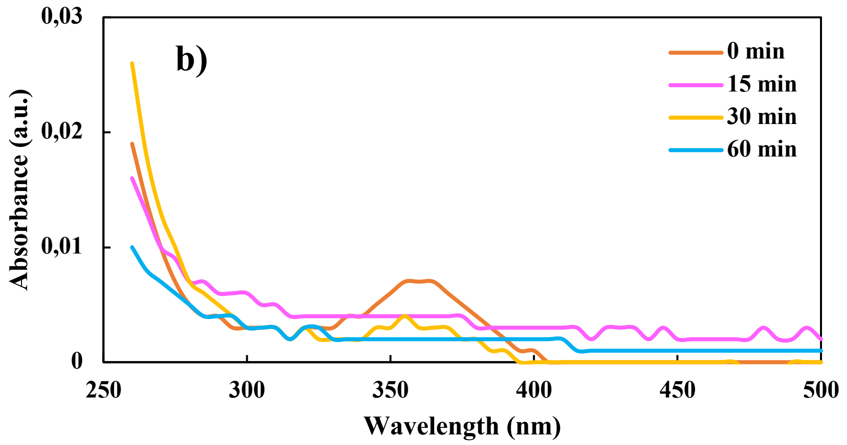

Fig. S11. UV-Vis absorption spectra of different H_2O_2 dosage during UV/H_2O_2 process: a) 0 µM H_2O_2; b) 1 µM H_2O_2 c) 2 µM H_2O_2 d) 5 µM H_2O_2 e) 10 µM H_2O_2.

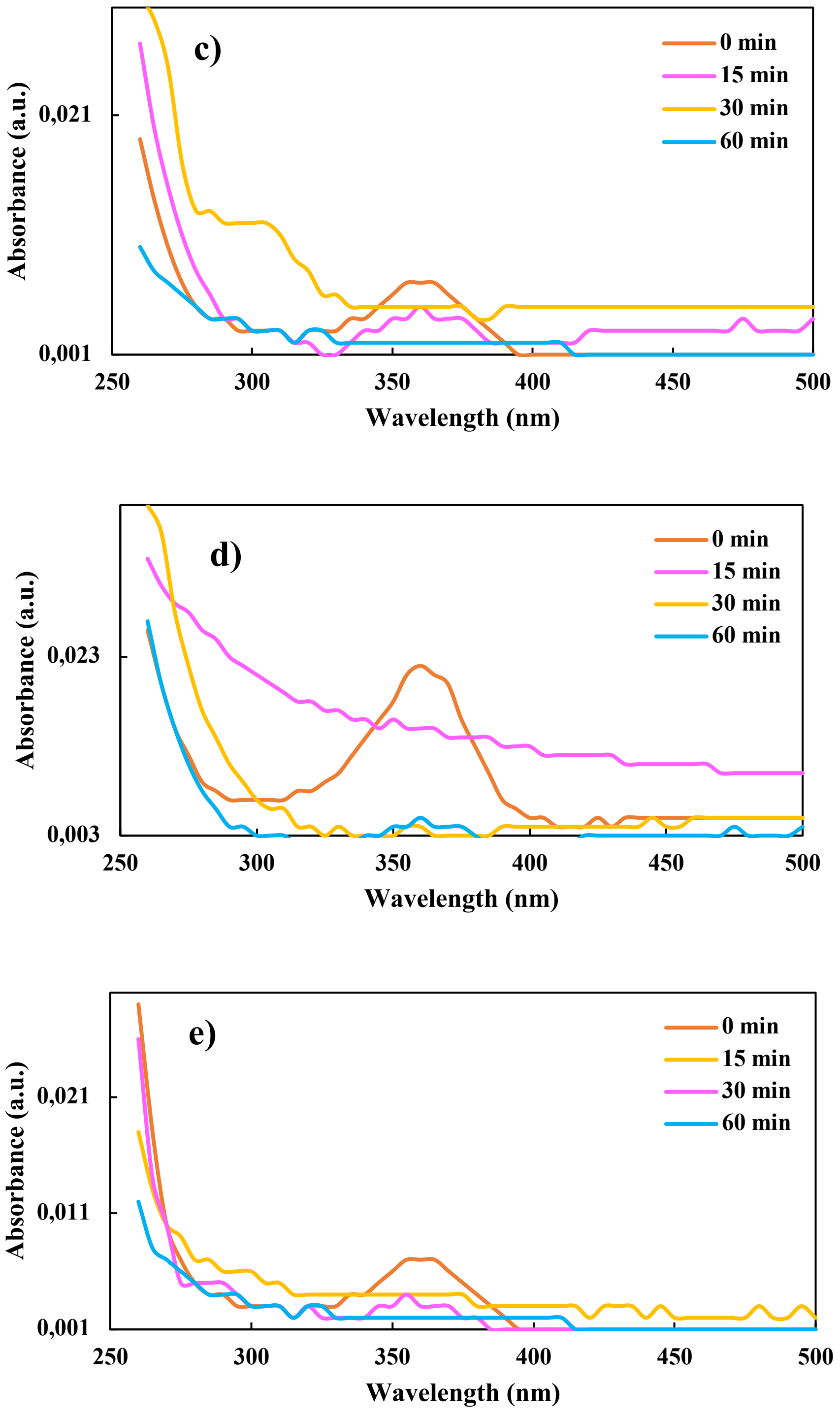

Fig. S11. *(continued).*

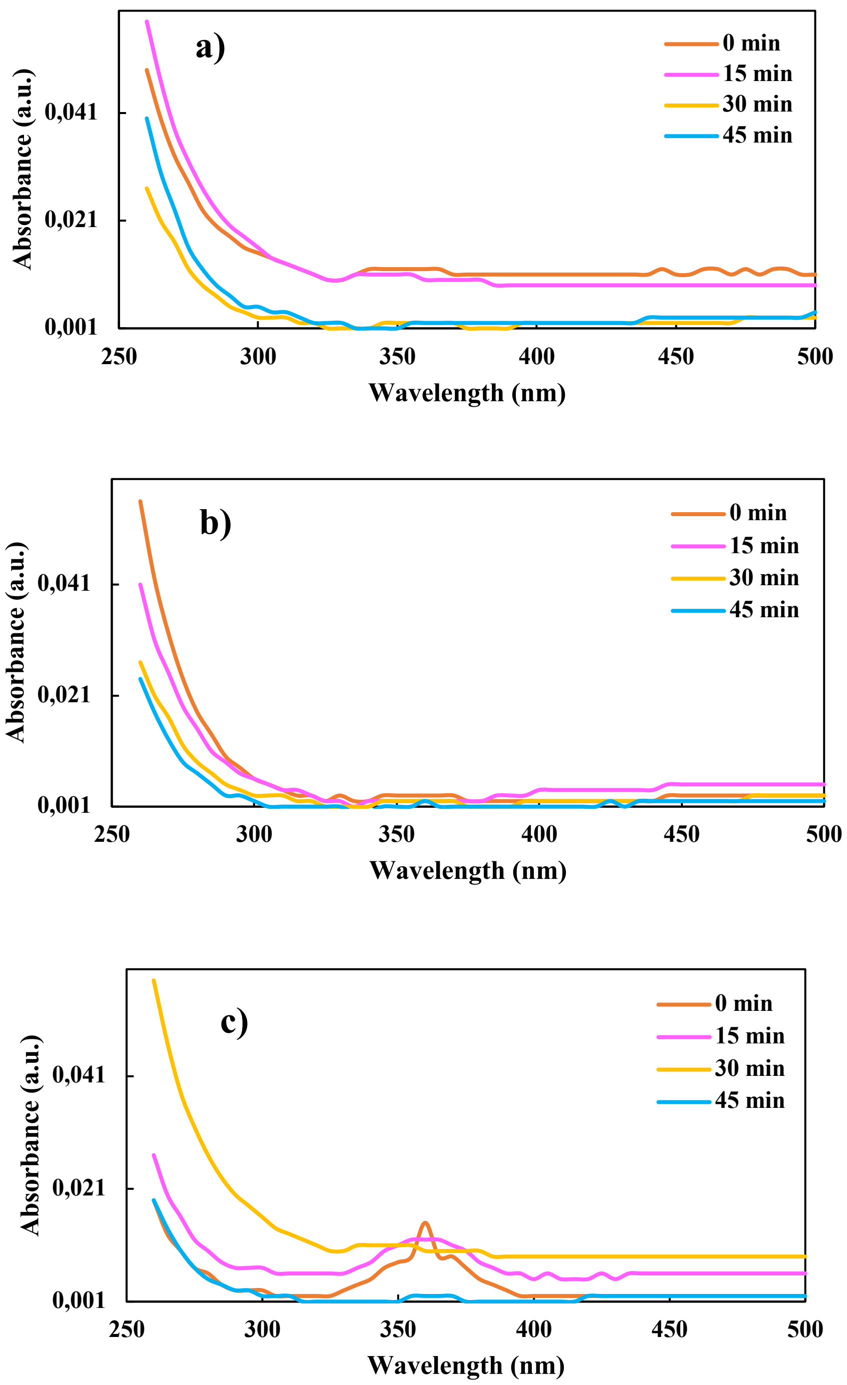

Fig. S12. UV-Vis absorption spectra of different pH dosage during UV/H_2O_2 process: a) pH 4 b) pH 5 c) pH 7 d) pH 9.

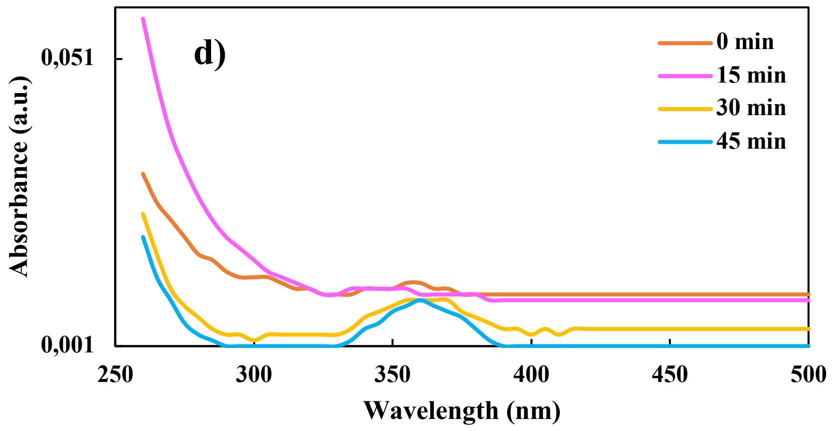

Fig. S12. *(continued).*

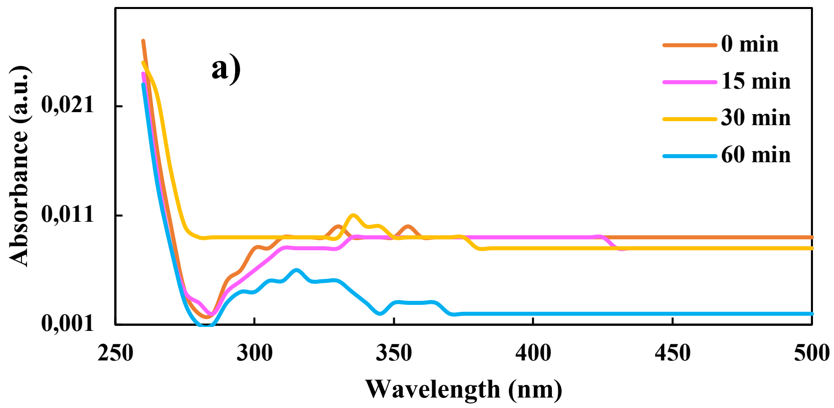

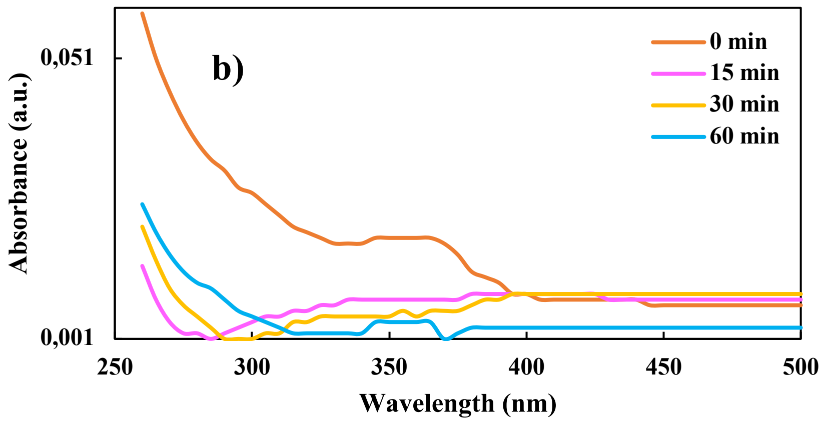

Fig. S13. UV-Vis absorption spectra of different water matrixes during UV/Co-doped ZnS QDs process a) DW b) TW c) WWTP.

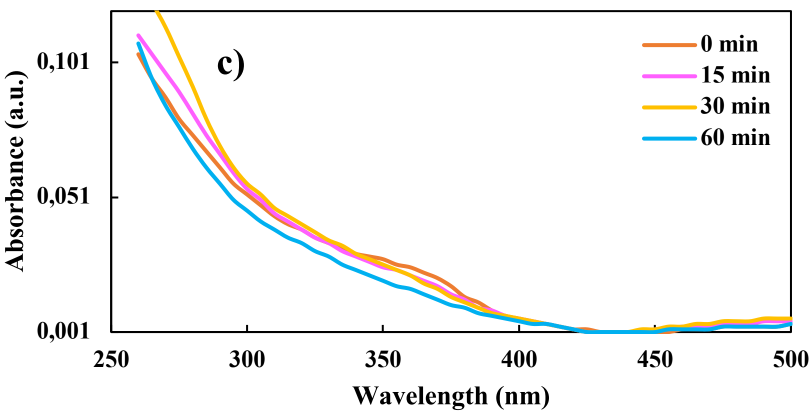

Fig. S13. *(continued).*

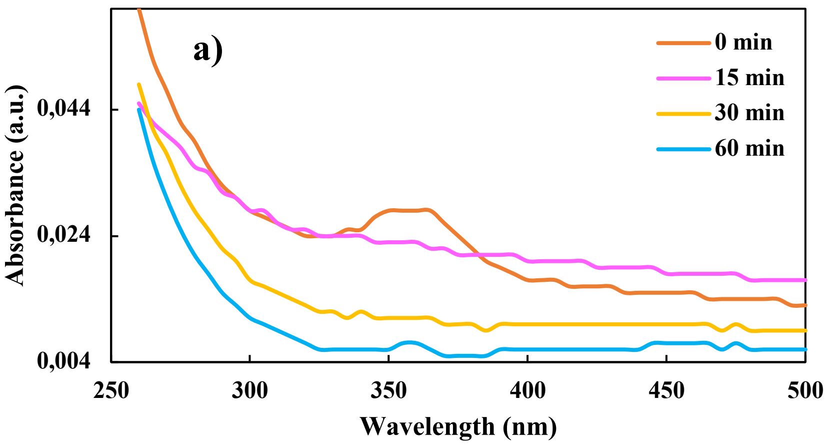

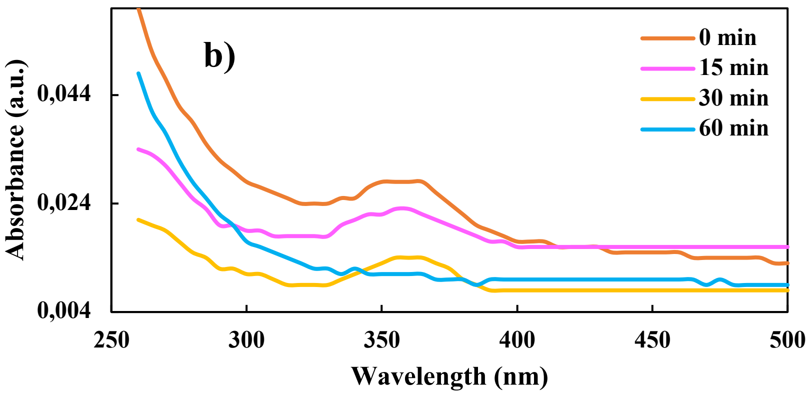

Fig. S14. UV-Vis absorption spectra of different catalyst concentrations during UV/Co-doped ZnS QDs process a) 5 mg/L b) 10 mg/L c) 20 mg/L d) 50 mg/L.

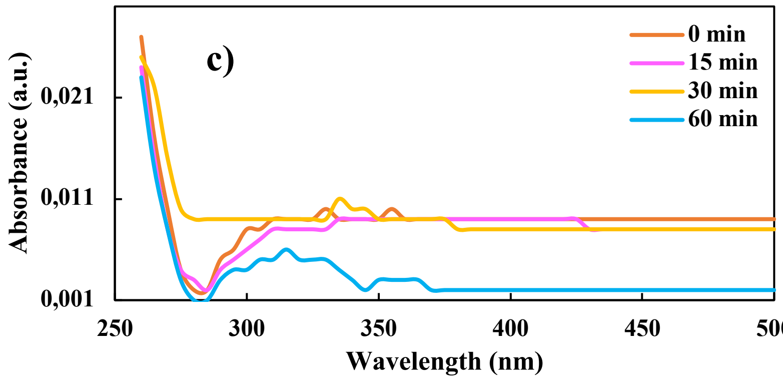

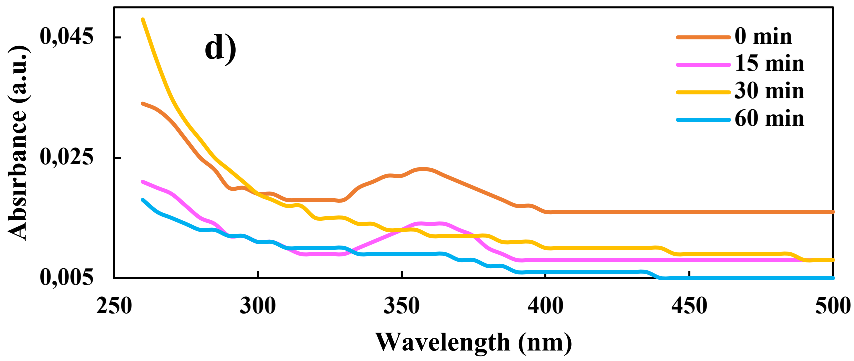

Fig. S14. *(continued)*.

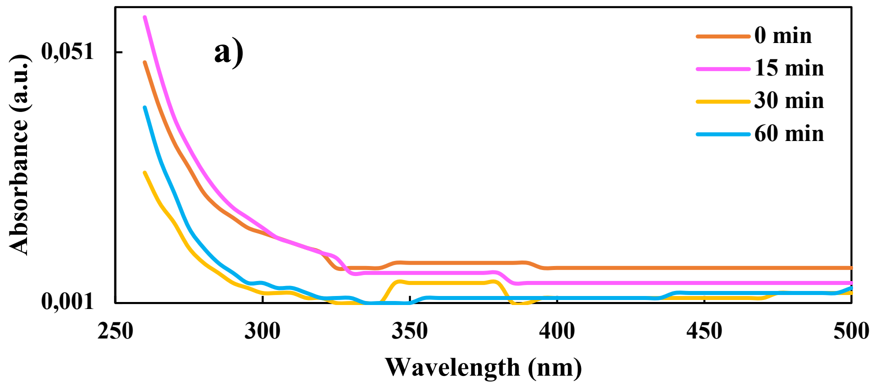

Fig. S15. UV-Vis absorption spectra of different pH dosage during UV/Co-doped ZnS QDs process : a) pH 4 b) pH 5 c) pH 7 d) pH 9.

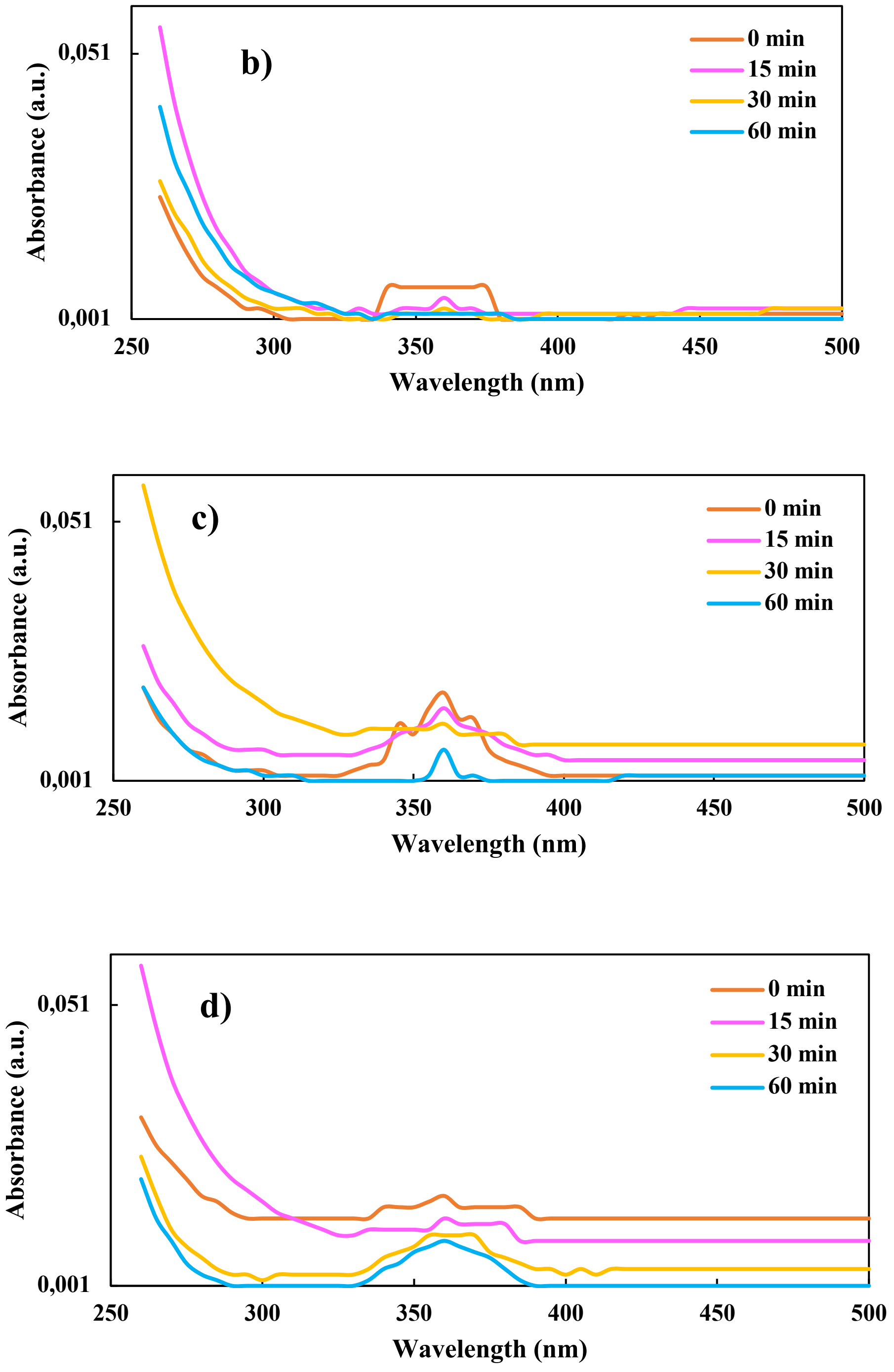

Fig. S15. *(continued).*

Table S1. The identified transformation products in FAV degradation by the UV process.

Compound	m/z	Molecular weight	Formula	Proposed Structure
Parent	156.02	157.02	$C_5H_4FN_3O_2$	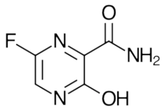
**Pathway I**				
1	188.95	189.96	$C_5H_4FN_3O_4$	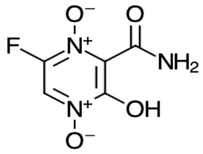
2	115.00	116.01	$C_4H_3FN_2O$	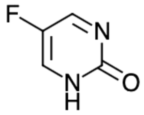
**Pathway II**				
3	138.97	139.97	$C_5H_4N_3O_2$	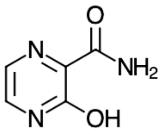
4	121.02	122.03	$C_5H_5N_3O$	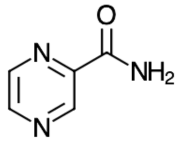
5	103.97	104.98	$C_6H_{15}N$	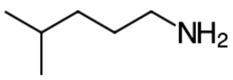

Table S2. The identified transformation products in FAV degradation by the UV/Co-doped ZnS QDs process.

Compound	m/z	Molecular weight	Formula	Proposed Structure
Parent	156.02	157.02	$C_5H_4FN_3O_2$	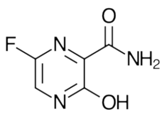
**Pathway I**				
1	138.97	139.97	$C_5H_4N_3O_2$	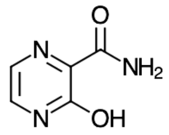
2	121.02	122.03	$C_5H_5N_3O$	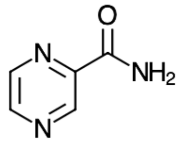
3	103.97	104.98	$C_6H_{15}N$	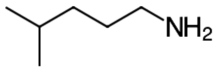
**Pathway II**				
4	140.98	141.99	$C_5H_4FN_3O$	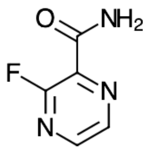
5	124.99	125.99	$C_5H_4FN_3$	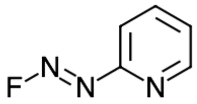
6	103.97	104.98	$C_6H_{15}N$	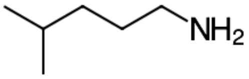

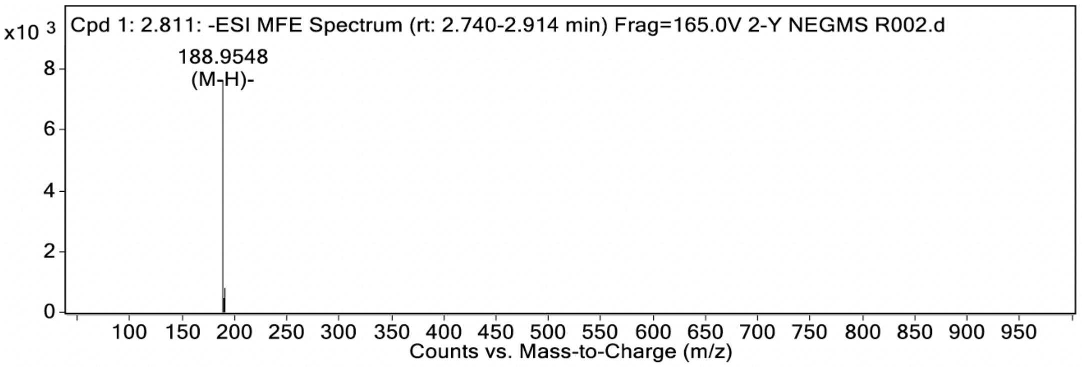

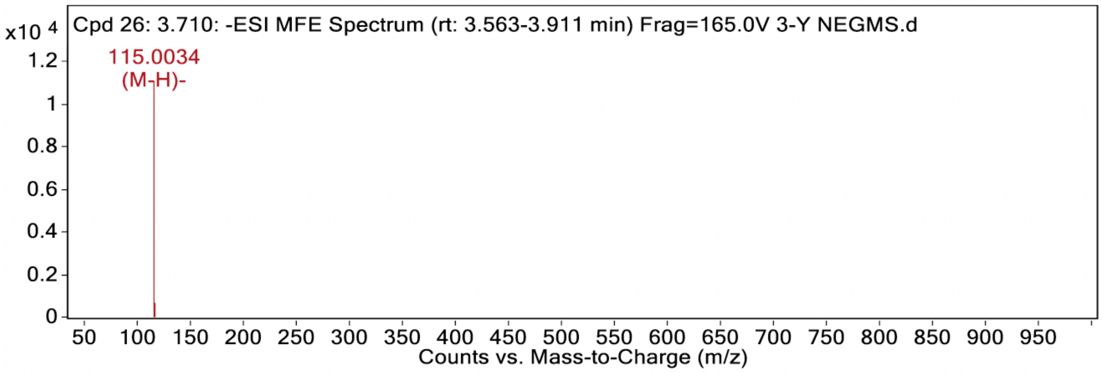

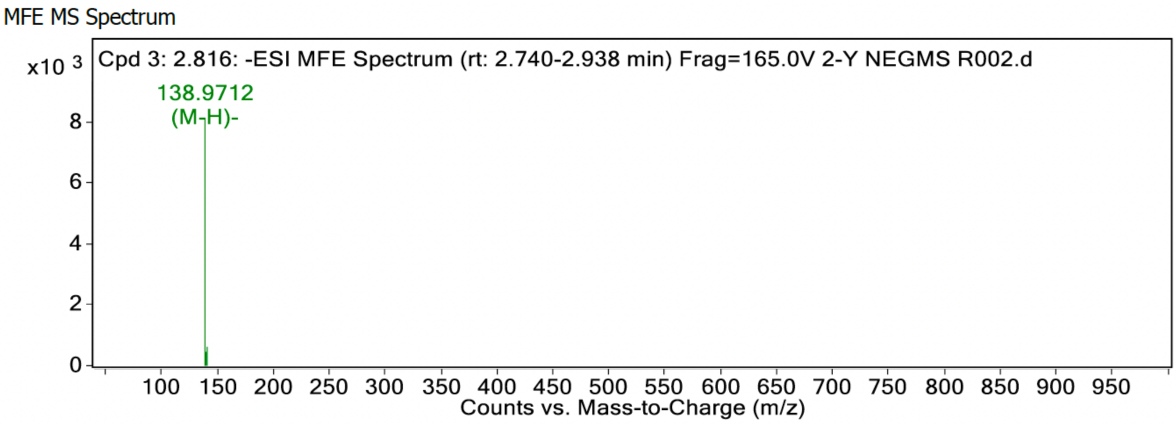

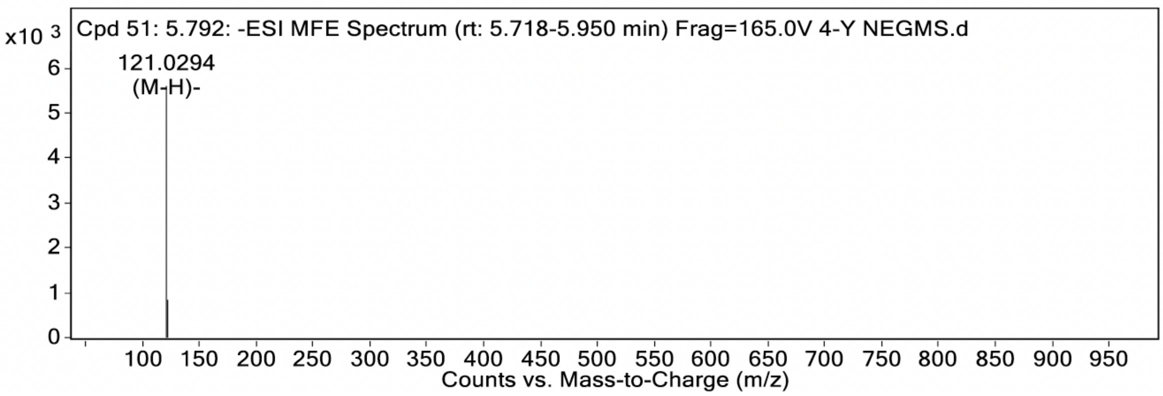

Fig. S16. LC-MS/QTOF spectra, at negative mode ($[M-H]^-$) of FAV degradation products.

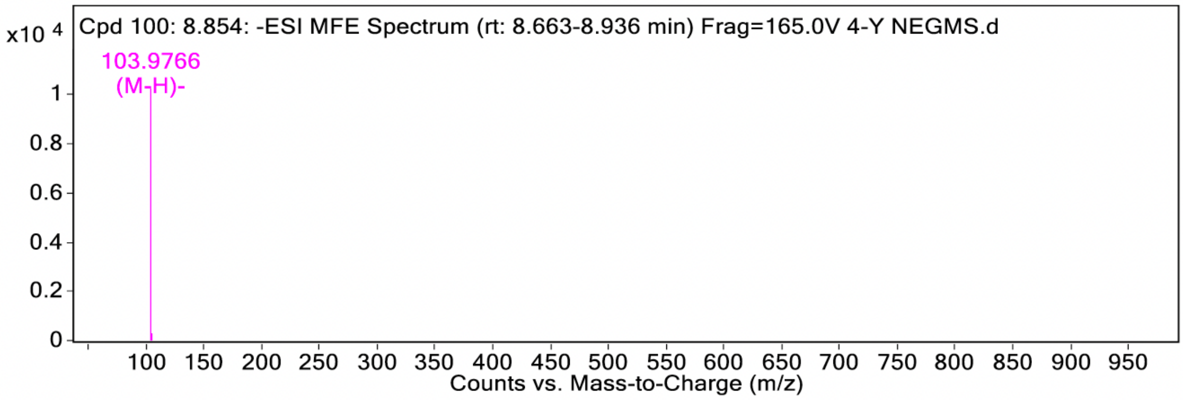

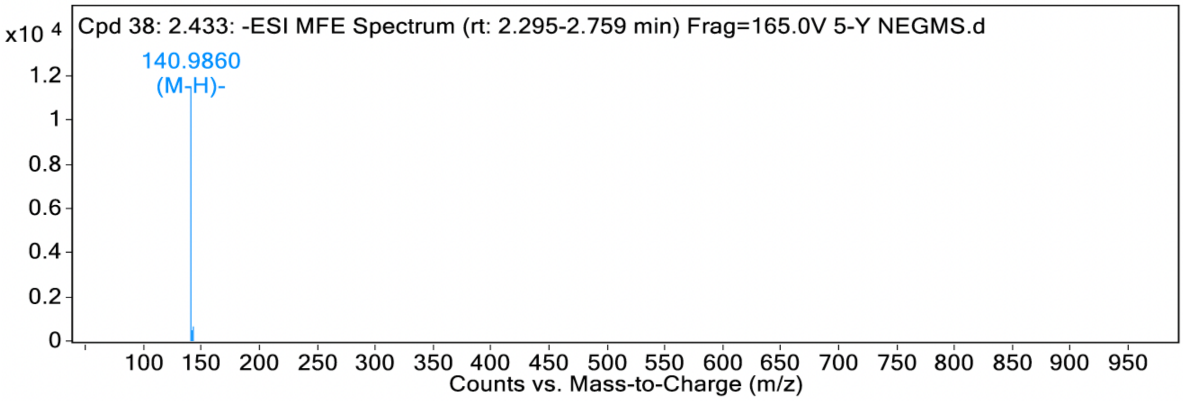

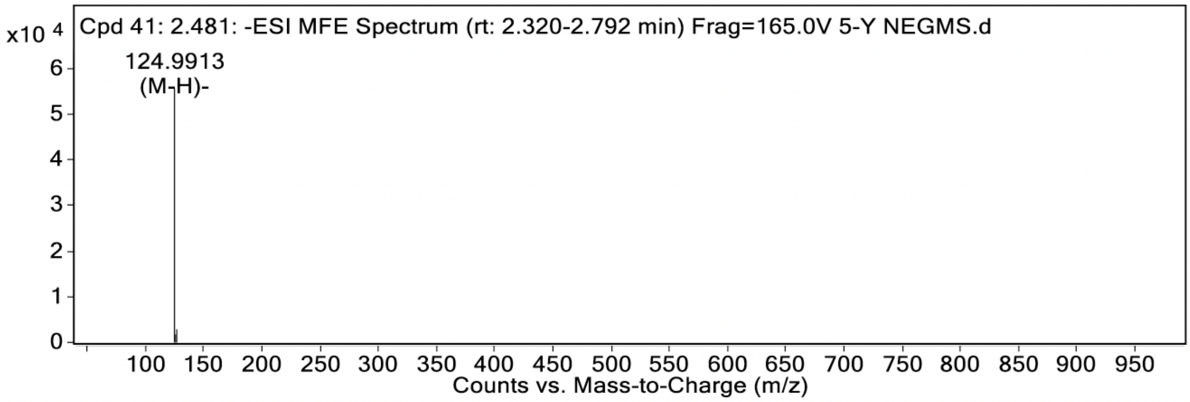

Fig. S16. *(continued).*
